# A Key Management Protocol Based on the Hash Chain Key Generation for Securing LoRaWAN Networks

**DOI:** 10.3390/s21175838

**Published:** 2021-08-30

**Authors:** Shimaa A. Abdel Hakeem, Sherine M. Abd El-Kader, HyungWon Kim

**Affiliations:** 1 School of Electronics Engineering, Chungbuk National University, Cheongju 28644, Korea; shimaakotb@cbnu.ac.kr; 2 Electronics Research Institute (ERI), El Nozha, Cairo 12622, Egypt; sherine@eri.sci.eg

**Keywords:** IoT communication, LoRaWAN security, hash chain generation, key updates issues, salt encryption, authentication, encryption

## Abstract

Recently, many Low Power Wide Area Network (LPWAN) protocols have been proposed for securing resource-constrained Internet of Things (IoT) devices with negligible power consumption. The Long Range Wide Area Network (LoRaWAN) is a low power communication protocol that supports message authentication, integrity, and encryption using two-session preshared secret keys. However, although the LoRaWAN supports some security functions, it suffers from session key generation and key update problems. This motivates us to introduce a new key management protocol that resolves the LoRaWAN problems and supports key updates. The proposed protocol is based on hash chain generation using a one-way hash function. Network entities share a common hash chain of *n* key elements to allow using a unique signing key per message. We also propose a salt hashing algorithm that encrypts the original keys into a different form to avoid the physical attacks at the end device side. We analyzed the proposed key generation performance in terms of the computation time, the required storage, and the communication overhead. We implemented and tested the proposed key generation protocol using the NS-3 network simulator. The proposed lightweight key generation protocol significantly enhances the security of the original LoRaWAN at a negligible overhead. The proposed protocol reduces the power consumption and transmission time by two times compared with some previous protocols. In addition, the proposed key generation protocol can resist attacks, such as key compromising attacks and replay attacks, and it supports the Perfect Forward Secrecy, which was not supported by LoRaWAN.

## 1. Introduction

Currently, it is the Internet of Things (IoT) era, where billions of tiny IoT end devices are maintained and deployed worldwide. According to an Ericsson report [1], the number of connected IoT devices has so far reached 28 billion in 2021. These IoT devices generate an incredible amount of data and transfer the collected information to different cloud servers, processed and accessed anywhere and at anytime. Many communication protocols have been proposed to support different types of IoT networks. Short-range communication protocols, such as ZigBee, Bluetooth, and Z-Wave have been used for utilizing the limited resources of IoT devices due to their low energy consumption [2].

However, these short-range protocols cannot be deployed for critical applications that require a wide communication range, such as smart cities [3]. Although cellular radio communication can provide long-range connectivity, IoT networks are not suitable due to their complexity and lack of cost-effectiveness [4]. Recently, Low Power Wide Area Networks (LPWAN) technologies have been proposed to satisfy the IoT constrained devices’ requirements and outperform previous conventional technologies’ shortcomings. They are maintained to enable a wide range of communication with low power consumption. Deploying these low power technologies can allow the battery-powered constrained sensors to transmit messages up to many kilometers and last for years [5]. However, many LPWAN exist, such as Lora, SigFox, Ingenu, Telensa, and Weightless, but only LoRa is considered the most effective cost design and low power consumption [6]. LoRa is a protocol that works at the physical layer to enable wide-range communication up to 15 km using chirp modulation [4]. At the same time, the upper layer of the LoRaWAN protocol is based on LoRa to define the operation of the system and the structure [7]. LoRaWAN is an asynchronous protocol that extends the battery lifetime by reducing the synchronization overhead.

A lot of IoT security protocols have been published to satisfy the requirements of tiny devices with limited storage and power. In [8], the authors provide an authentication scheme for multi-group communication based on bilinear pairing. However, they prove that this method consume more power and require extra storage which makes it not suitable for IoT networks. In [9,10], the authors proposed an authentication technique based on a hash chain to support decentralized key generation and message authentication using the Message Authentication Code for vehicular communication. In [10], they prove as well how a hash chain is secure and the computation cost is negligible, which makes it suitable for IoT devices. The authors of [11] present a Secure Sensor Cloud Architecture (SASC) for IoT networks to support data efficiency and improve network security and scalability. Another related work that presents a comprehensive investigation of different authentication schemes for mobile devices is presented in [12]. The authors of [13] proposed a security analysis of access control and authentication for the IoT networks and applications. However, although we analyzed many IoT authentication protocols, they still suffer from high power consumption and complexity. Recently, some related authentication protocols have been proposed mainly for low power, wide area networks.

Most of the existing LPWAN protocols mainly focus on power issues and communication range issues, while security was a secondary issue that did not have an excellent investment. The security importance in the IoT networks becomes more critical than previously because many threats are related to peoples’ real lives. Moreover, the loss from security events can be severe due to the effective connectivity and scale of the IoT networks. Earlier research on IoT security [14,15] has discussed some significant factors; one is key management. According to the studies, security keys can be sniffed by several attacks, considering that IoT devices are usually deployed wherever the attacker can reach them. Some security studies proposed a solution to prevent key sniffing and defend against physical attacks. In [16], the authors proposed a lightweight solution for securing data provenance in IoT networks using Fingerprints. They used physical unclonable functions (PUFs) and wireless fingerprints that are defined from the wireless channel information to achieve data anonymity, provenance, and mutual authentication. Leaking key material from the end devices is considered a great issue in IoT networks.

LoRaWAN protocol specifications [17] indicate that the session keys and root keys should be managed to reduce the key compromising damage. Whatever keys are leaked from an end device, the other network devices continue the communication safely without affecting their security. However, still, the LoRaWAN suffers from the session key updates that are considered a critical issue, and at present, there has been no proper solution. However, LoRaWAN utilizes security keys for various security mechanisms, such as message authentication and message encryption; the current LoRaWAN protocol slightly updates these keys. In some situations, an end device must keep using specific keys for its lifetime. Thus, if the key is leaked in the future, all the data transferred between the end devices may be compromised by the attacker. Therefore, keys must be renewed regularly to prevent the key compromising attacks.

In LoRaWAN protocol v1.02 [17], it is pointed out that compromising the session keys of one end device cannot impact the secure communication of the other end devices. Though, in the Activation by Personalization mode, the session keys are generated using the end device address, which results in vulnerability by reverse engineering.

Further, the original LoRaWAN protocol weakens the end-to-end security and cannot resist replay attacks. The authors of [18] proposed some attacks that affect the data integrity, network availability and data confidentiality in the previous versions of LoRaWAN. The authors highlighted the replay attacks and desynchronization attacks. These attacks consider the network server or the end device as the target entity. The authors discuss two techniques for the replay attack: (1) Join-Accept message replay attack and (2) Join messages harvest.

Furthermore, the authors consider the end device or network server as the target for the desynchronization attacks that can disconnect the end devices from the network. The authors also recommended that the AppNonce support freshness provides the detection against the replay attacks. In addition, they recommend verifying the received Join-Accept message that matches the sent Join-Request message and checking if the session keys have been shared or not. The authors of [19] attempted to implement end-to-end security by allowing the AppSKey negotiations between the end device and the application server without including the network server. However, this needs the changing of the original LoRaWAN, making it challenging to apply it in the existing LoRaWAN standard. Furthermore, this method cannot support the perfect forward secrecy.

In this paper, we propose a key management protocol that supports the generation of the hashed key using one master secret key that is managed by the network server. The hash chain generation is a lightweight solution to support unique keys per session and support periodic updates for the session keys. We also propose salt encryption for the generated keys to protect the original key from attackers. Physical attacks in the original LoRaWAN can happen as the end devices can be reached easily, and the key material can be accessed. The proposed protocol supports two cases, the first one for low power applications with high security and the second one for extremely low power applications with a medium security level. The proposed protocol enhances the key update mechanism by using n keys for future communication. Storing these keys with salt encryption can hide the original value and can prevent any physical attacks. Any compromising for a salted key can expose any information about the original hidden key and cannot compromise the original master secret key generated by the network server per each end device for future communication between the end device and network server.

The proposed protocol uses the AES protocol in two modes for authentication and encryption and can support a high-security level over the original LoRaWAN. The power consumption in the proposed two cases is slightly different from the original LoRaWAN that consumes lower power than the proposed protocol, due to its communication overhead. The contributions of this paper are summarized as follows:

- Proposing a dynamic hash chain key generation that produces short-lived session keys. The use of distinct hash-based random keys for authentication and integrity check can increase the security level and avoid attacks that compromise the key;
- Proposing salt encryption for the hashed keys to prevent the physical attacks by the self-generation of random values that can hide the original keys from the outside attackers and inside attackers;
- Preventing replay attacks by adding a timestamp to each transmitted message;
- Supporting low-overhead key updates to further enhance the security level and prevent the key compromising attacks;
- Supporting forward secrecy, which means that even if a past key was compromised, it would not allow the attacker to predict future keys;
- Supporting secure key exchange between the network server and the end devices (encrypting the server’s messages using the stored session keys).

This paper is organized as follows: Section 2 describes the previous LoRaWAN studies that explain the key management problems of LoRaWAN. Section 3 presents the LoRaWAN security background. Section 4 describes the proposed protocol architecture. The proposed protocol security analysis is discussed in Section 5. The security verification using AVISPA is provided in Section 6. The performance evaluation and communication overhead analysis are presented in Section 7. In Section 8, the conclusions and future work are provided.

## 2. Related Work

In [20], a security report provides the potential vulnerabilities of LoRaWAN and the fundamental classification of LoRaWAN security. According to this report, all entities in LoRaWAN could be compromised during the key management process, the inter-communications, and the Internet connection to the application and network servers. In [21], the author analyzed the DevNonce generation of the LoRaWAN. The DevNonce is included in messages as a random value generated by the end device to avoid replay attacks. The author analyzed the method mathematically and concluded that the end device could be unavailable with a confident probability using the system’s current DevNonce.

To mitigate this problem, the author suggested extending the size of the DevNonce value to 32 bits. In [22], the authors propose a scheme for securing LoRaWAN networks; their method uses the proxy node concept. In general, the proxy nodes estimate the trustworthiness of each other to build a table and transmit it to the end node. Finally, the end node communicates over the most trusted proxy node with the highest trust value. The original LoRaWAN protocol has key generation and key update problems. The key updates problem has not been discussed in any LoRaWAN security study. In [23], the authors proposed a solution that solves the session key generation problem, but it has some downsides. The authors suggest adding a trusted third-party entity to support session key generation; however, the added third party makes the join procedures more complex and increases the communication overhead.

Another problem with the LoRaWAN protocol is that the network server generates both session keys. Thus, the network operators can decrypt and capture all the data passing the network server. The authors of [23] also have analyzed this problem and recommended deploying a secure third party to enhance the security of the LoRaWAN network.

The most critical security problems of the original LoRaWAN and previous methods are summarized as follows:

- Single point of failure: The network server generates the session keys per session for each end device that can affect network security if the network server is compromised;
- No perfect forward secrecy: The LoRaWAN system depends on the preloading of two root keys used to generate the session keys. If the long-life root keys (AppKey, NwkKey) are compromised, the previous session keys can be recovered. Therefore, the original LoRaWAN cannot satisfy the perfect forward secrecy;
- Physical attacks: Long-term keys and session keys are accessible to the attackers since they are stored in flash memory at the end device. The original LoRaWAN cannot resist the physical attacks;
- Network operators vulnerabilities: Once the network server generates the application key AppSKey for the application server, the network operators can decrypt the end device data and modify it. Separation of the network server role and application server role is required;
- No key update in the OTAA: Join procedure in the Over-The-Air Activation (OTAA) mode using the long-term keys (NwkKey and AppKey) to derive the session keys. The generated session keys can be updated by sending a new Join-Request message. However, the long-term root keys cannot be updated. These keys are preloaded at each end device by the manufacturer. The root key update is critical for the devices that join the LoRaWAN network via OTAA;
- No key update in the ABP: The AppKey and NwkKey are not preloaded on the end device in the Activation By Personalization (ABP) mode. Only session keys (AppSKey, NwkSKey) are stored to allow immediate communication between the end device and the network server. The end device uses the same session keys for a lifetime. If the end device is physically attacked, the session keys are compromised, posing security threats at the end device.

We summarize the differences between the proposed protocol and the other existing methods in Table 1. Some defined security requirements must be satisfied to enhance the security level of each protocol, such as mutual authentication, message integrity, message encryption, session key updates, and secure key exchange. Moreover, each security method must have a defense against the well-known attacks (e.g., physical attacks, replay attacks). All mentioned protocols in Table 1 support mutual authentication via Over-The-Air Activation, message integrity using AES128-CMAC, and message encryption using AES128-CTR. The key update is supported only for [23] and the proposed protocol, while for the other protocol, only two longlife keys are used for different sessions. In the proposed protocol, session key updates are supported, as each end device uses a unique key per session. There is no defense against the key attacks for protocols [18,19,20,22], as the root keys and session keys can be sniffed and derived. In contrast, the proposed protocol is able to defend against the key attacks using a salted key-table that hides the original keys in a different encrypted form. Hiding keys prevents attackers from sniffing them or even compromise. There is no defense against the replay attacks for protocols [19,20,22], as the end device does not register the received AppNonce in the Join-Accept message. However, the proposed protocol can defend against replay attacks by attaching a fresh timestamp with each message. Perfect forward secrecy is supported only for [19,22] and the proposed protocol, in which the compromising of the initial root keys cannot compromise the encrypted session keys. In the proposed protocol, using salt encryption hides the original keys in a different form that prevents any type of key attacks. In this paper, we propose a key management protocol based on a hash chain generation. The proposed protocol supports key updates and solves the session key generation problems without including other trusted third parties. To avoid compromising the end devices from the radio operators, each end device can self-generate random numbers to encrypt each session key and only send these random values for the authorized receivers to generate the new session key that can be used to authenticate the received messages.

## 3. Background of LoRaWAN Protocol

### 3.1. Architecture of LoRaWAN

**Figure 1:**
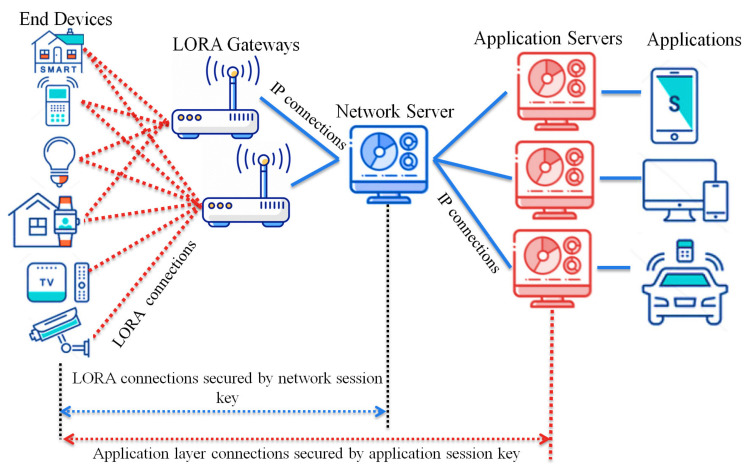
Network architecture of LoRaWAN.

LoRaWAN protocol [24] is designed for limited battery applications where wide-range communication with low power consumption is primary. LoRaWAN v1.02 specification defines the network ranges to be (5–15) km; data rates range between 0.3 and 50 kbps, and the network is operated over the 868-MHz and 900-MHz bands. LoRaWAN is one of the essential technologies for IoT that has grown based on the network star topology. Its structure tries to grant interoperability between IoT devices regardless of their properties. The LoRaWAN architecture consists of the following entities: end device, gateway, network server, and application server. As shown in Figure 1, each end device can be connected to many gateways over a single network hop where the gateways are connected to the network server over IP connections. LoRa radio connections are used to support communications between the end devices and the gateways, while the network servers and gateways are connected using IP communication. We briefly describe LoRaWAN elements:

1. End device: An IoT end device is used for wide-range communication to transfer small data using low-frequency bands. These end devices can be deployed in different fields, such as smart building, factory automation, smart cities, and farm automation;
2. Gateway: An IoT end device with high capabilities to receive data from the end nodes via a LoRaWAN radio link. Then it forwards the collected packets to the corresponding network server via standard IP communication;
3. Network server: It is a LoRaWAN server that controls and manages the whole network. It receives many packets, removes redundancy between them, executes authentication checks to accept or reject the packets, and finally decides which gateway is the most suitable to send an acknowledgment packet back to the end device. In our paper, we consider the Network Server as a trusted third-party agent that is authorized by a Certificate Authority (CA) organization to generate and execute security tasks, manage key generation, and support revoking mechanisms for the malicious nodes. To reduce the communication burden on the Certificate Authority (CA) due to the frequent security message requests of the end devices gateways.

The network server authenticates the LoRa messages using the network session key shared previously between the end device and the network server. The LoRaWAN protocol stack consists of the MAC and application layers, while the physical layer uses the LoRa RF. The application layer communication is secured with the application session key shared previously between the application server and the end device. The message format of the original LoRaWAN consists of the MAC payload, MHDR (MAC header), FHDR (frame header), and MIC (Message Integrity Code). The MIC is calculated over the MAC payload using the network session key for authentication purposes. The MAC payload can be a Join-Request message or Join-Accept message, while the size varies between 51 and 222 bytes.

### 3.2. Security Aspects of LoRaWAN

New end devices must complete an activation process to join the LoRaWAN network. Two session keys are shared between the end devices and the network server during this activation process. LoRaWAN protocol has two types of activation. The first type is Over-The-Air Activation (OTAA), and the second type is Activation by Personalization (ABP) [25].

#### 3.2.1. Over-the-Air Activation

The end devices communicate with the network server to start the network joining procedure. In [26], the activation mode is used when the end device is initially deployed. Initially, each end device is configured with root keys (NwkKey, AppKey), which are configured by the manufacturer. The network key (NwkKey) and the application key (AppKey) allow the end device to derive the future communication session keys.

**Figure 2:**
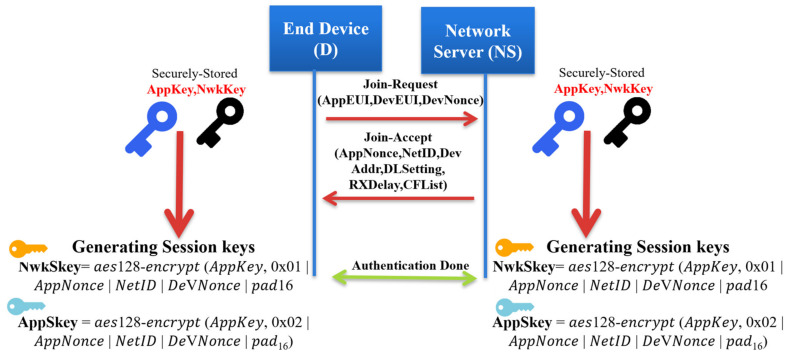
Over-The-Air Activation in LoRaWAN networks.

Figure 2 shows the operation of the Over-The-Air Activation of an end device in a LoRaWAN network. We explain each step in detail as follows.

Join-Request message: The end device begins the network joining procedure by transmitting a Join-Request message. Each end device includes the application identifier (AppEUI), the global end device address (DevEUI), and the random value (DevNonce) in the Join-Request message. The MIC value is calculated over the Join-Request message to ensure message authenticity and integrity. The following formula calculates the MIC code using the AES 128 protocol in the authentication mode:

(1) $$\mathrm{AES}128\;\mathrm{CMAC}\;(\mathrm{NwkKey},\;\mathrm{MHDR}\;\left|\left|\;\mathrm{AppEUI}\;\right|\right|\;\mathrm{DeVEUI}\;||\;\mathrm{DeVNonce})\;\;$$

**Figure 3:**
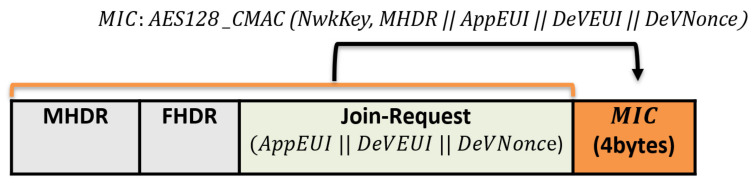
The Join-Request message structure.

The NwkKey is the network root key previously preshared between the end node and the network server (configured by the manufacturers or at commissioning). The Join-Request message format is shown in Figure 3.

Authentication at the network server: The network server performs the security check over the received Join-Request message by checking the freshness of the attached DevNonce to avoid the replay attack. If the DevNonce is not valid or previously used, the network server rejects the joining request. Otherwise, the network server completes the end device authentication process by calculating the MIC over the received Join-Request message. Comparing the calculated MIC with the received MIC to authenticate the end device validity, if both are equal, the network server starts generating the session keys. Both the Network Session Key (NwkSKey) and Application Session Key (App SKey) are derived using Equations (2) and (3) as follows:

Nwk SKey = aes128 − encrypt (NwkKey, 0x01||AppNonce||NetID||DeVNonce||pad16)(2)

(3) $$\mathrm{App}\;\mathrm{SKey}=\mathrm{aes}128-\mathrm{encrypt}\;\left(\mathrm{AppKey},\;0x02\;\left|\left|\;\mathrm{AppNonce}\;\right|\right|\;\mathrm{NetID}\;\left|\left|\;\mathrm{DeVNonce}\;\right|\right|\;\mathrm{pad}16\right)\;$$

The network server generates an AppNonce random number. NetID refers to the network ID and separates the geographically duplicated LoRaWAN networks.

**Figure 4:**
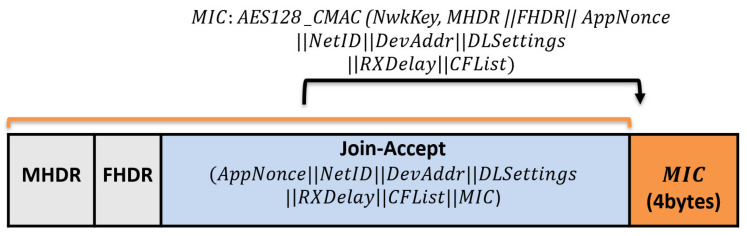
The structure of Join-Accept messages.

Join-Accept message: it contains NetID, AppNonce, DevAddr, RxDelay, DLSettings, and CFList. The Device Address DevAddr is a 32bits that is assigned for the end device within the joined network. RxDelay represents the delay between the sending and receiving process. DLSettings represents the configuration of the downlink. The optional field CFList represents the channel frequencies. Once the end device receives the Join-Request message, it encrypts the parameters using AES-128 to generate the network session key, the NwkSKey and the application session key AppSKey. The Join-Accept message structure is shown in Figure 4.

#### 3.2.2. Activation by Personalization

Each end device can join any LoRa network in ABP mode without the Over-The-Air activation or any join procedures. Thus, the end device does not need AppEUI, DevEUI, NwkKey, and AppKey, which are crucial components for Over-The-Air activation. In contrast to the OTAA mode, the session keys (NwkSKey, AppSKey) and device address (DevAddr) are pre-stored on the end device in the ABP mode. Therefore, each end device can instantly start communication once it is powered.

## 4. The Proposed Protocol

**Figure 5:**
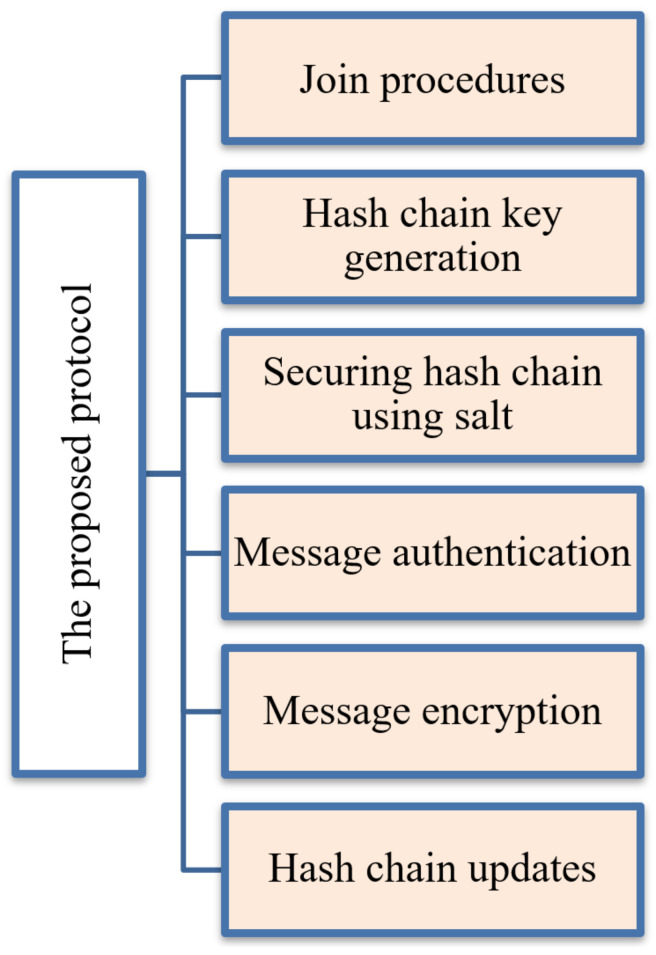
The proposed protocol architecture.

Each end device node communicates with the network server for establishing session keys for message authentication and encryption. The frequent communications between nodes and network servers increase power consumption, increase communication costs, and cause network congestion. The current security problems of the LoRaWAN systems motivate us to propose an enhanced, lightweight key management protocol that allows end devices to save power and reduce communication costs. This section describes the proposed protocol with the following steps—Join procedures, securing the generated hash chain against physical attacks, message authentication, message encryption, and hash chain updates. Figure 5 summarizes the proposed protocol architecture that introduces the key generation method. Table 2 summarizes the mentioned variables and system notations.

### 4.1. Join Procedures

As mentioned before, the LoRaWAN security systems are based on a pre-shared key structure. The Application Key (AppKey) and the Network Key (NwkKey) are used to generate two session keys (NwkSKey and AppSKey). In both join modes OTAA and ABP, the session key updates are considered as a problem. Once the end devices in the ABP mode cannot update the session key, it requires the AppKey and NwKwy to generate new session keys. Only long-term session keys are preloaded at each end device for long life in the ABP mode. In contrast, in the OTAA mode, the key session updates depend on the long-term root keys that can be compromised at any time. Due to these problems, we propose a lightweight key management protocol that enhances the security level and reduces power consumption.

Our proposed key generation method uses the initial communication OTAA mode to join the LoRaWAN network and share the security parameters with the network server for future session communication. Like the original LoRaWAN, each end device sends a Join-Request message, while the server responds with a Join-Accept message; both entities derive the session keys for the current session, as explained in Section 3. Finally, the network server shares the application session key AppSKey with the application server, as illustrated in Figure 2.

**Figure 6:**
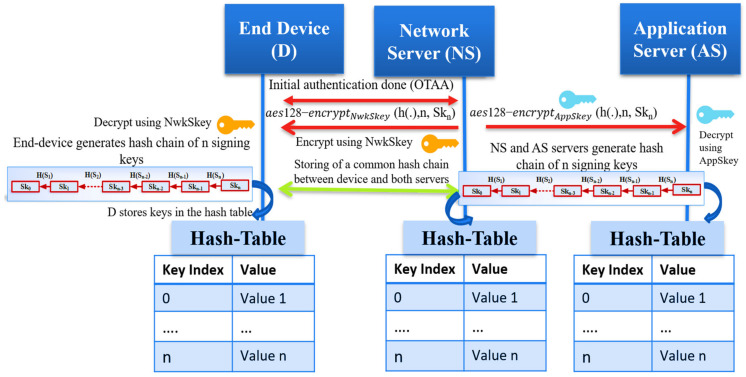
The proposed hash chain generation in LoRaWAN networks.

After the OTAA Authentication between the network server and the end device is complete, both share a common one-way hash function h(.) of length n, a master secret key Sk_n_ that is generated by the network server. By iteratively hashing Sk_n_, the end device generates n keys using the cryptography function h(.) of length n. We allow each device to generate n unique signing keys from the shared master secret key. The proposed solution helps the end device avoid using the long-term root keys to update the session keys as the original OTAA mode. Also, it prevents the use of preloaded, long-term session keys as ABP mode. The following section explains the key generation method at the end device and the network server. We also summarize the proposed hash chain generation in LoRaWAN in Figure 6.

### 4.2. Hash Chain Key Generation

We assume the trustiness of the network server that makes it works as a Key Distribution Center; the network server picks a random number to represent the seed (Sk_n_) for the hash function. After the end device passes the Over-The-Air Authentication phase, the network server securely sends the master secret Sk_n_ to the end device and the application server (AS) to generate the typical hash chain. A one-hash function h(.) is defined as z = h(y) for a given input y. It is challenging to predict the primary input y from the hashed output z. Hash chains are first proposed by Lamport [27]. Recently, it is enhanced to support the one-time password systems by utilizing the hash function h (·) to generate a hash chain of length n starting with a seed (Sk_n_). The output result of this iteratively hash generation is a set of unique keys. The output hashed elements are considered session keys to secure future communication between the end device and both servers (NS, AS).

We summarize the key generation as follows:

- Through the OTAA mode, the network server shares two session keys (NwkSKey, AppSKey) with the end device and application server, respectively;
- The network server (NS) securely shares the key material message that consists of (one-way hash function h(.), the length n of the chain, the master secret seed Sk_n_ to generate the chain);
- The NS transmits the encrypted key material message for both the end device and the application server securely;
- Both the end device and application server decrypt the key material message using the NwkSKey and AppSKey, respectively, previously shared with the network server;
- All entities start generating a typical hash chain using the hash function h(.) and Sk_n_;
- The session keys can be regularly updated and changed based on a policy between the end devices and the network server;
- The hash chain keys are indexed and securely stored in a key table using the Hardware Security Module (HSM);
- The used hash function is sha256 that generates 256-bits; only 128-bits are used as AES keys for authentication and encryption.

### 4.3. Securing the Hash Chain Using SALT

A critical problem in the original LoRaWAN is the physical attacks against the end devices that compromise the long-term keys. The compromising of the long-term keys affects the generated session keys and the whole network system. The prestored hash table at the end devices is protected using the salt technique that prevents the exposure of the session keys. Salts are random numbers self-generated by the end devices after the initial authentication to the LoRaWAN network. The end device randomly generates salt values to hide the actual original secret keys in an encrypted form.

**Figure 7:**
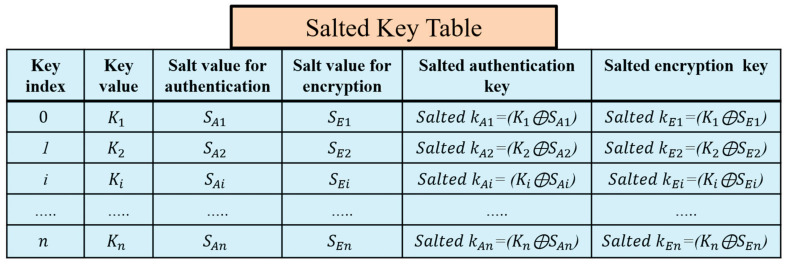
The self-generation of authentication and encryption salted keys at the end device.

Salts are used to preserve the passwords in storage. Salting a key is the XORing operation of this key, and a random number results in a new key that can be stored safely with the salt value without holding the actual key. Our proposed key generation prevents any physical attacks using the salt technique for authentication keys and encryption keys. The salted key table is shown in Figure 7. The end device erases the key table after the key generation completes and keeps the salts values and the salted authentication and encryption keys. Salts numbers do not require to be separately stored from the hashed passwords or to be encrypted. If the attacker accesses the hashed table of the salted keys and salt values, the attacker cannot predict the original keys. Using self-generated salts at the end devices protects them from the network operators’ attacks and protects the original session keys from being compromised.

### 4.4. Message Authentication

The end device node randomly picks a secret key from the prestored hash chain key table to calculate the Message Integrity Code (MIC) over the message. This MIC is considered as a checksum to prevent the tampering of messages. LoRaWAN utilizes the AES-CMAC algorithm to calculate the MIC value over a message to support authentication and integrity. AES-CMAC uses the Advanced Encryption Standard [NIST-AES] as a building block. AES-CMAC uses a secret key and a variable-length message as inputs to return a fixed-length string as MIC. Our proposed protocol uses the key index only to inform the receiver which key to calculate the MIC over the message. Sending the index of the signing key and a random salt value allows the receiver to find the salted authentication key value. By XORing, the received salt value with the original signing key generates the salted authentication key. Sending only the key index and salt value prevents key compromising attacks; even if the attacker sniffs the index, it cannot expose any information about the originally key. Conventional MIC algorithms require sending the signed key and the MIC value calculated over a message to allow the receiver to verify the message. The proposed algorithm does not require sending the key to improve the security level. Both the end device and server store the same hash chain table of n key elements. Each sender randomly chooses one salted authentication key to sign the message using AES128-CMAC and attaches the index of the key; the salt value is used to hide the authentication key to the message as a pointer to the key. The proposed protocol can significantly improve the computation speed, increase the security level, and reduce the network overhead.

The message authentication procedures are as follows: 

The end device picks a salted authentication key $\mathrm{Salted}\;k_{\mathrm{Ai}}$ from the salted key table to generate a message integrity code over a data message m, where $K_{i}$ is the original key, and $S_{\mathrm{Ai}}$ is the salted value. The authentication procedure is described through Equations (4)–(6).

(4) $$\mathrm{Salted}\;k_{\mathrm{Ai}}=\left(K_{i}⊕S_{\mathrm{Ai}}\right)$$

(5) $${\mathrm{MIC}}_{\mathrm{Salted}\;k_{\mathrm{Ai}}}=\;\mathrm{AES}128-\;\mathrm{CMAC}(\mathrm{Salted}\;k_{\mathrm{Ai}},\;m)$$

where

(6) $$m=\left(T_{s}\left|\left|k_{\mathrm{index}}\right|\left|S_{\mathrm{Ai}}\right|\right|m_{\mathrm{Di},\mathrm{NS}}\right)$$

The parameters of Equation (6) are described as follows:

${\mathrm{MIC}}_{\mathrm{Salted}\;k_{\mathrm{Ai}}}$: The MIC value for message m using a salted authentication key $\mathrm{Salted}\;k_{\mathrm{Ai}}$.
$T_{s}$: The current timestamp to avoid replay attacks.
$K_{\mathrm{index}}$: The pointer of the used key in authentication in the pre-shared common hash table
$S_{\mathrm{Ai}}$: The random generated salted value is used to hide the authentication key.
$m_{\mathrm{Di},\mathrm{NS}}$: The transmitted message from the end device Di to the network server Ns.

The sender Di attaches the obtained ${\mathrm{MIC}}_{\mathrm{Salted}\;k_{\mathrm{Ai}}}$, $S_{\mathrm{Ai}}$ and the $k_{\mathrm{index}}$ of the signing key and the timestamp, Ts, to the transmitted message as depicted in Figure 9. When the server NS receives the message $\left(T_{s}\left|\left|k_{\mathrm{index}}\right|\left|S_{\mathrm{Ai}}\right||m_{\mathrm{Di},\mathrm{NS}}\right||{\mathrm{MIC}}_{\mathrm{Salted}\;k_{\mathrm{Ai}}}\right)$, NS checks the freshness of the timestamp, Ts. If Ts is invalid, NS rejects the message; otherwise, NS verifies the ${\mathrm{MIC}}_{\mathrm{Salted}\;k_{\mathrm{Ai}}}$ value of the received message. NS queries the stored hash table using the received key index, $k_{\mathrm{index}}$. Then it XORings the key value with the received salt value $K_{i}$⊕$S_{\mathrm{Ai}}$. The XORing output represents the salted authentication key $\mathrm{Salted}\;k_{\mathrm{Ai}}$ that is used by the sender to sign the message.

Then, it calculates the message integrity code of the received message, ${\mathrm{MIC}}_{\mathrm{Salted}\;k_{\mathrm{Ai}}}$ as in Equation (7).

(7) $${\mathrm{MIC}}_{\mathrm{Salted}\;k_{\mathrm{Ai}}}^{*}=\mathrm{AES}128-\mathrm{CMAC}\left(\mathrm{Salted}\;k_{\mathrm{Ai}},\;m\right)\;$$

If the calculated ${\mathrm{MIC}}_{\mathrm{Salted}\;k_{\mathrm{Ai}}}^{*}$, is equal to the received one, ${\mathrm{MIC}}_{\mathrm{Salted}\;k_{\mathrm{Ai}}}$, the network server NS accepts the message.

### 4.5. Message Encryption

In the original LoRaWAN, the network server (NS) shares some parameters with each end device to derive the AppSKey using these parameters and the prestored AppKey. After the application session key generation, the NS securely transfers the AppSKey to the application server (AS).

In our proposed protocol, the NS sends the h(.), n, and the Sk_n_ to the application server to start generating the same hash chain for the end device, as shown in Figure 6. The application server securely saves the hash chain of n keys for future encryption of the application layer messages. The encryption of messages between the end device and application server is conducted using AES-128 encryption. The transferred messages between the end device and application server are entirely secure, which prevents neither the gateway nor the network server from reading it. We propose a salt random number generator at the end device to hide the original key and encrypt it. Then it uses the salted encryption key to encrypt the message and sends the index of the key and the salt value for encryption to the AS. These parameters allow the AS to derive the salted encryption key by XORing the received salt with the original hashed key value and decrypt the messages. When the end device wants to send a message m to the application server, it randomly picks an element from the hash chain table and XORings this key with the salt value for encryption. The salt and salted encryption key values are stored at the end device side, as shown in Figure 7.

The message encryption procedures are as follows: 

At the end device side: Picks a salted encryption key ${\mathrm{Salted}\;k}_{\mathrm{Ei}}$ from the salted key table to encrypt a message m, where

(8) $${\mathrm{Salted}\;k}_{\mathrm{Ei}}=\;\left(K_{i}⊕S_{\mathrm{Ei}}\right)$$

$K_{i},$ represents the original key from the hash table and ${S\;}_{\mathrm{Ei}}$, represents the salt number used for the generation of the salted encryption key ${\mathrm{Salted}\;k}_{\mathrm{Ei}}$.

(9) $${\mathrm{Em}}_{\mathrm{Di},{\mathrm{Salted}\;k}_{\mathrm{Ei}}}=\;\mathrm{AES}128-\;\mathrm{encrypt}\;\left({\mathrm{Salted}\;k}_{\mathrm{Ei}}=\left(K_{i}⊕S_{\mathrm{Ei}}\right),\;m\right)$$

${\mathrm{Em}}_{\mathrm{Di},{\mathrm{Salted}\;k}_{\mathrm{Ei}}}$, represents the application encrypted message m using the salted $k_{\mathrm{Ei}}$.

- After encryption, the end device sends the message, salt value, and key index to the application server.
  (10) $$(T_{s}\;\left|\left|k_{index}||Em_{Di,Salted\;k_{Ei}}\right|\right|S_{Ei})\;$$
- The application server checks the timestamp $T_{s}$ to ensures the message freshness and using the key index as a pointer to the hash table to find the original key $K_{i}$.
- It XORings the key value $K_{i}$ with the received salt value $S_{Ei}$, $K_{i}$⊕$S_{Ai}$, the result represents the $Salted\;k_{Ei}=(K_{i}⊕S_{Ei})$ that used to decrypt the received encrypted message $Em_{Di,Salted\;k_{Ei}}$.

Using salt increases the hashed key’s security, making it hard for an attacker to compromise the used key and prevents the application server from tracking the end device data. To prevent physical attacks on the end devices, we recommend that each end device store only the salted hashed keys and the used salted values.

## 5. Security Properties

In this section, we discuss the proposed key management security properties and objectives compared with the original LoRaWAN security protocol:

### 5.1. Session Key Distribution

The network server is considered a trusted third-party agent in our proposed solution to generate the master key for the end devices. It securely shares a master secret seed value with the end device and application server. All entities (end device, network server, and application server) use a one-way hash function to generate n hashed keys that can be used for message authentication and encryption. Instead of frequent communication with the network server and application server for deriving new session keys or new session key updates, as in the basic LoRaWAN solution, our proposed key management protocol allows the end devices to securely store the hash chain of n elements using a Hardware Security Module. This solution enhances the security level and prevents the use of two long-life session keys as previous LoRaWAN. Our protocol needs only a one-time Over-The-Air Activation to share a session key between the end device and the network and application server. In the initialization phase, the end devices use the pre-shared session keys (NwkSKey and AppSKey) to share a new master secret key Sk_n._ The end device iteratively hash the Sk_n_ to generate n hashed keys used as future session keys instead of using the NwkSKey and AppSKey for the long lifetime. In order to avoid the physical attacks at the end devices side, we propose a salt solution to hide the original session keys, so the end devices only store the salted keys and the salt numbers in the database. If an attacker tries to compromise the stored salted keys, he is unable to expose any information about the original session keys. Our proposed solution allows the end devices to send the key index value as a pointer of the original key that is prestored at the network server and end devices instead of sending the key over the open wireless medium as previous authentication solutions for more security. We sacrifice the storage needed to keep an n hash length of keys at the communicated parts to enhance the security level and optimize the network bandwidth. 

### 5.2. Session Key Updates

**Figure 8:**
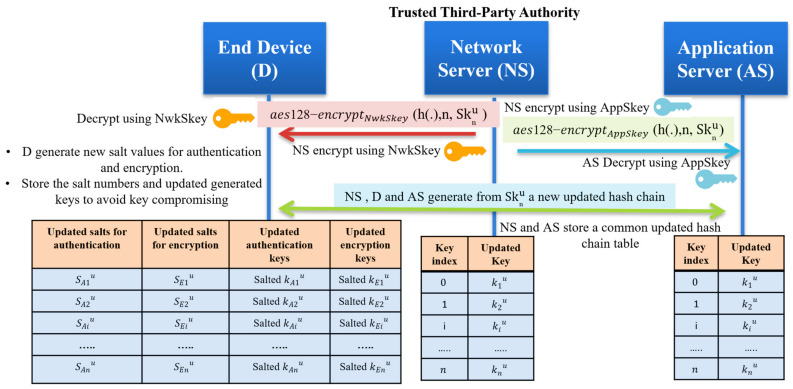
The master secret key update and self-generation of new salted keys for authentication and encryption.

In the basic LoRaWAN security protocol, no key updates happened, which is considered a critical issue. The end devices are using two session keys for a long lifetime for authentication and encryption. Our protocol solves the session key update problem by providing different unique keys every session. The end devices store n keys securely; each key is valid for a short time. Randomly choosing one key from the prestored keys and sending only the key index allows the receiver system to authenticate the message. To enhance the security level of the prestored hash chain, we recommend updating the master secret key Sk_n_ to generate a new hash chain based on an agreement between the network server and the end device. The session key updates can be achieved as follows:

- The network server generates a new master secret $Sk_{n}^{u}$;
- Securely send the $Sk_{n}^{u}$ for both the end device and application server using the pre-stored root keys (NwkSKey and AppSKey);
- All communicated entities generate a new hash chain of length n, then the network server and application server storing the generated hash-chain;
- The end device salted the generated hashed chain with random numbers to store new encrypted keys and only different salt numbers;
- The end device stores only the salted authentication keys and sated encryption keys in addition to the updated salted values. The hash chain key updating process is shown in Figure 8.

### 5.3. Mutual Authentication

The proposed protocol uses the OTAA join procedure of the original LoRaWAN to authenticate the end device node to the network server. After the mutual authentication is complete, the network server shares a secret key to allow the end device to generate the keystream of the hashed elements for future session communication. Each end device stores a length n of hashed keys, n key indices, n salted values for authentication, and n salted values for encryption. The original LoRaWAN allows devices to use two lifetime keys to derive the network session keys and application session keys. However, the proposed protocol allows devices to use different salts for authentication and encryption, which increases the security level of the session keys.

### 5.4. Secure Key Exchange

The proposed hash chain key generation includes transferring the master secret key Sk_n_ in an encrypted way using the NwkSKey that prevents any attacker from exposing the initial master secret. In contrast to the original LoRaWAN, the key derivation depends on the initial parameters that are transferred between the end device and network server in the Join-Request message and the Join-Accept message. The generation of session keys requires transmitting a new request message to the server, which results in significant overhead at the network server and high power consumption at the end device side. The proposed key generation method does not use the long-term keys for session key generation. However, using the root keys to derive the session keys in the original LoRaWAN exposes the generated session keys for being compromised once the root keys are compromised. Key generation depends on the master secret key generated by the network server. Storing n keys for future communication provides updated session keys that are picked randomly to sign and encrypt the messages.

### 5.5. Defense against Key-Compromising Attacks

In the original LoRaWAN, all session keys are generated using the root keys that make them vulnerable to sniffing attacks. However, the end device generates a keystream from the initial seed key that is preshared securely between the end device and network server in the proposed key generation method. The end device randomly picks any key from the stored salted hash table that hides the original keys in an encrypted form called salted keys. If any attacker physically accesses the end device, the stored key information is unable to reflect the original key parameters (initial seed used to generate the hash chain elements).

### 5.6. Perfect Forward Secrecy

Perfect forward secrecy is a security property where the compromise of long-term (root) keys does not expose the past derived session keys [28]. The proposed key generation depends on the master secret key Sk_n_ securely shared between the end device and network server. This key generates n hashed keys by iteratively hashing it n times using one-way hash functions (sha256). Once the proposed key generation method does not depend on the long-term keys to generate the session keys, finding the past session keys is difficult by compromising the root keys. The end devices store only the encrypted version of the original hashed keys (salted keys) to avoid sniffing the original keys shared and stored at both the network and application servers. Thus, the proposed key generation supports perfect forward secrecy and backward secrecy.

Backward secrecy means that the compromise of the long-term keys cannot affect the generation of the future session keys. The proposed protocol allows the end devices to pick randomly one session key for authentication and then send only the key index to allow the receiver to verify the message. Sniffing the key index does not disclose any information about the key, which guarantees forward secrecy.

### 5.7. Defense against the Replay Attacks

For the original LoRaWAN, the network server registers the DevNonce included in the uplink messages (Join-Request) to prevent replay attacks. However, the end device for the downlink messages (Join-Accept) does not register the received AppNonce from the network server. Therefore, an attacker may register a (Join-Accept) message, wait for the end device to send another (Join-Request) to respond with the registered (Join-Accept). In this case, the end device and the network server derive different session keys. Our proposed key generation method includes a timestamp in each uplink and downlink transmitted and received message to prove the freshness of the messages and prevent any attacker from transmitting different faked messages. Therefore, we proposed to use timestamps instead of nonces to protect the end device and the network server from replay attacks. We summarize the security properties comparison between the proposed protocol and the original LoRaWAN in Table 3.

## 6. Security Verification

In this section, we provide formal security analysis and automatic security verification for the proposed key management protocol and the authentication procedures using AVISPA. Automated Validation of Internet Security Protocols and Applications (AVISPA) is a formal security analysis tool that is used for modeling, design, and verification of security protocols. The High-Level Protocol Specification Language (HLPSL) is used to model the security protocol and then convert it to Intermediate Format (IF) using HLPSL2IF. The converted Intermediate Format is analyzed via the other four sub-modules, and the verification result is derived [29,30]. The four sub-modules are (1) On-the-Fly Model-Checker (OFMC), (2) SAT-based Model-Checker (SATMC), (3) CL-based Attack Searcher (CL-AtSe), (4) Tree-Automata-based Protocol Analyzer (TA4SP). For more information concerning the AVISPA modules, the reader can refer to [30]. SPAN is the security protocol animator that is used to build the protocol’s message sequences, implementing active intruders and different attacks.

**Figure 9:**
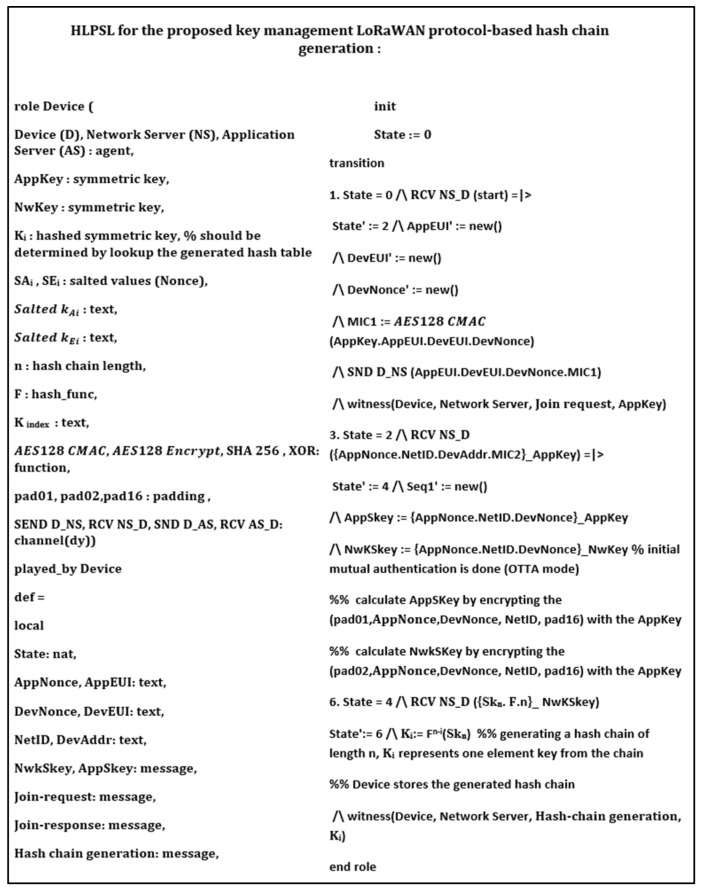
End device role using HLPSL and AVISPA.

**Figure 10:**
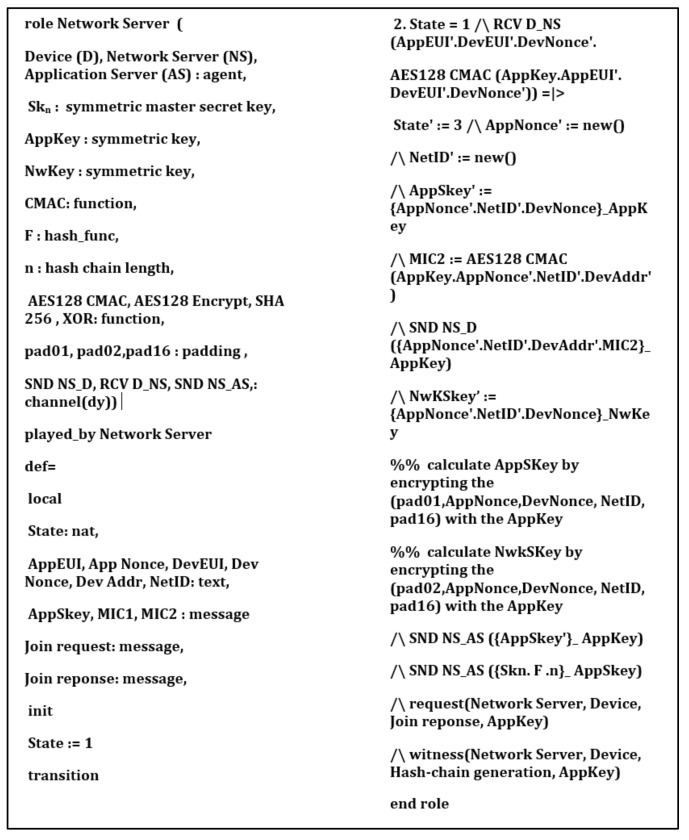
Network server role using HLPSL and AVISPA.

**Figure 11:**
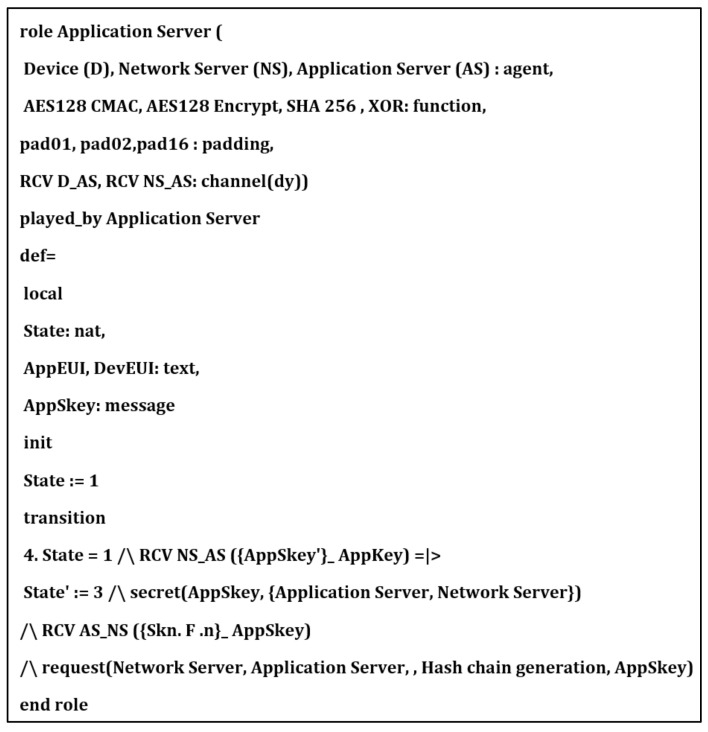
Application server role using HLPSL and AVISPA.

**Figure 12:**
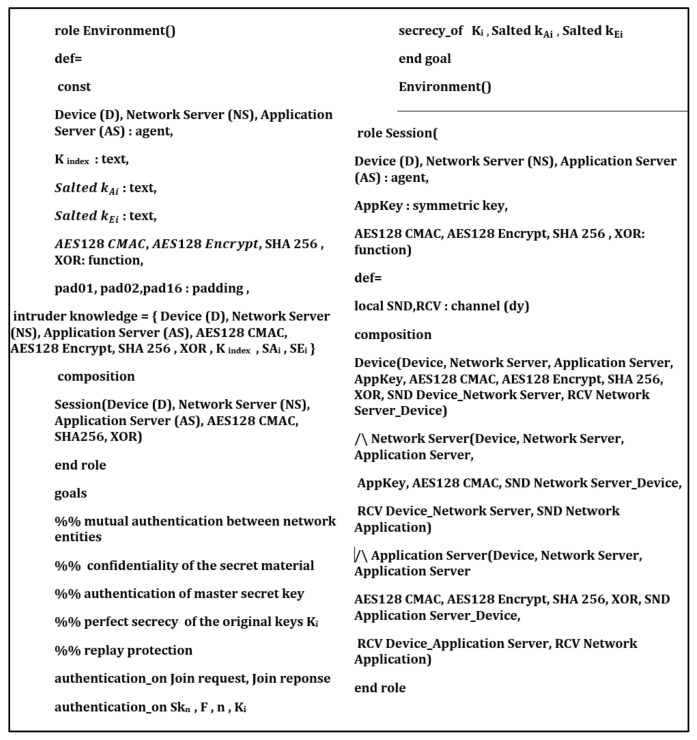
Environment and session roles using HLPSL and AVISPA.

The proposed key management protocol is designed in HLPSL and consists of three different roles (basic, composed, environment). The basic role includes three roles corresponding to three network entities, the Device (D), the Network Server (NS), and the Application Server (AS). We used AVISPA verification to model all network entities and the messages between them. Moreover, the AVISPA checks the security features of the proposed protocol and its ability to defend against some attacks. We define some security goals and intruder knowledge, session parameters, and environment. The network entities’ roles are described in Figure 9, Figure 10, Figure 11 and Figure 12, as defined in AVISPA.

**Figure 13:**
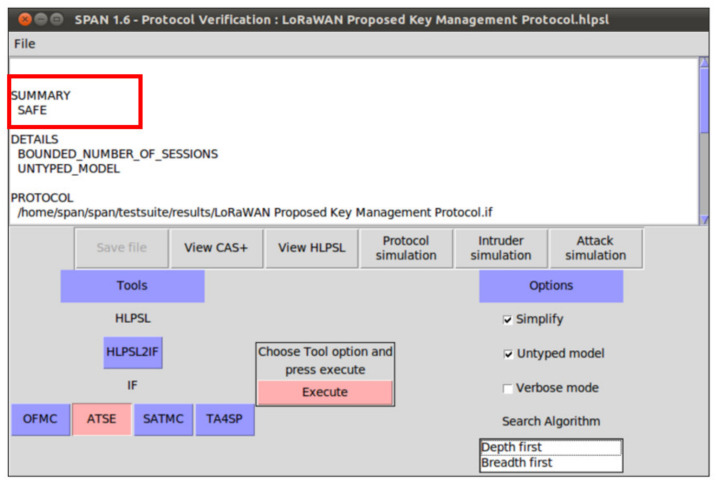

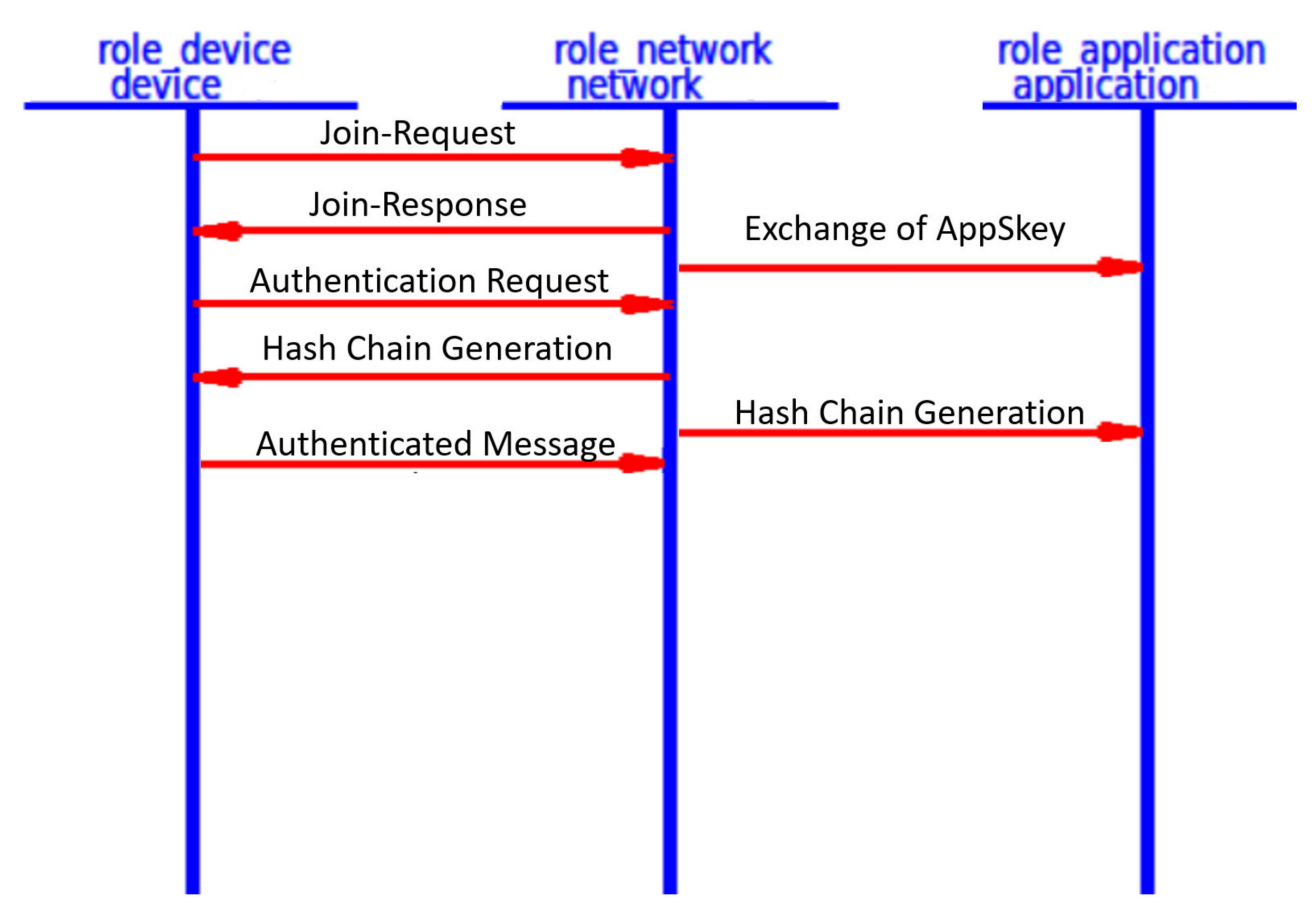
The proposed protocol security verification and message sequence using SPAN.

The following security properties and attacks are verified and discussed: mutual authentication, perfect forward secrecy, replay attack, unforgeability, and stolen verifier attack. The verification results using AVISPA confirm that the proposed protocol can support the previously mentioned security functions and resists the replay attack, unforgeability, and the stolen verifier attack. The ATSE module confirms that the proposed protocol is safe based on the defined security goals and the environment parameters. The security verification and message sequence of the proposed protocol are illustrated in Figure 13.

Security Verification Discussion:

We introduced a common hash chain of n keys that are stored only at the authenticated and authorized network entities (network server, end devices, application server). The signing keys used are based on the previously shared master secret and one-way hash function. These parameters are shared between the end device and the network server in an encrypted way to prevent any unauthorized user from intercepting or sniffing them. Any node that joined the network must start with the Over-The-Air Activation mode to do the mutual authentication and share two session keys one key for authentication (network session key), and a second key for encryption (application session key). If any end device receiver passed the initial mutual authentication, it can sign messages, generate valid signatures, and verify signatures. In our protocol, only registered and authorized devices can send the authenticated messages. If the receiver (network server) receives an incorrect signature, initially it calculates the signature over the message and starts comparing the received signature with the calculated signature; if it is not equal, the network server will discard the message. Therefore, the proposed protocol is secure concerning unforgeability.

According to the definition of the Stolen Verifier Attack, an attacker can steal the verification data from the server to generate communication data using the stolen verification information and sending it to the server. If it succeeds, the attacker can impersonate the end device information from the next authenticated session. However, in the proposed protocol, each end device generates a unique random number called salt authentication value that XORing it with the original secret key, then stores only the salted results and salted values in the salt table. This proposed salt encryption prevents the key compromising attacks for the end device side and also the server side. When the end device sends a message for the server, it sends a key index value that pointed to the originally used key, the salted value, timestamp, and device address. The end device never sends the actual key, it sends only the index of the used key. Only the server and end devices have the common hash table, so the server starts to search the table for the used key, picks up the key, and XORings it with a received salt value; the result is the key used by the end device to generate the message integrity code signature over the message. The server then compares the received signature with the generated signature and decides to accept the message or reject it. Now, even the network server is unable know the used key without the generated salt value by the end device, which prevents the network server attacks and preserves the end device’s security.

Moreover, if any attacker passed to steal the verification data from the server side, it can only control the current session. However, the future sessions are still secure as the end device uses a unique key per session. Therefore, the proposed protocol defends against the Stolen Verifier Attack. Each LoRaWAN end device is embedded with the tamper-proof device (Hardware Security Module), which prevents an attacker from accessing the security material and parameters. Moreover, the stored keys are stored in different forms using salt encryption, which is recommended for hash table security.

If anyone gets access to the hardware module, which is impractical, the attacker cannot return the salted keys to the originally used keys, which makes it very difficult for the attacker to sniff the keys or sniff the master secret key that is used to generate the total hash chain. Concerning forward secrecy, which is a security property where the compromise of long-term (root) keys does not expose the past derived session keys, the proposed key generation depends on the master secret key (Skn) that is securely shared between the end device and the network server. This key generates n hashed keys by iteratively hashing it n times using one-way hash functions (sha256). Once the proposed key generation method does not depend on the long-term keys to generate the session keys, finding the past session keys is difficult by compromising the root keys. The end devices store only the encrypted version of the original hashed keys (salted keys) to avoid sniffing the original keys shared and stored at both the network and application servers. Thus, the proposed key generation supports perfect forward secrecy. Even the end devices did not send the keys, so sniffing of the key index has no meaning and cannot disclose any information about the future session keys or past session keys.

## 7. Performance Evaluation

After discussing the security analysis of the proposed key generation protocol, we evaluated the proposed solution in terms of computation cost and communication cost. We also compared it with the basic LoRa security solution and some other related work that is proposed to enhance the security level of the Lora architecture. To evaluate the performance of the proposed protocol, we implemented it in an NS3 simulator using a cryptography library called MIRACL and a LoRaWAN module [31,32]. The simulations were conducted in a hardware platform employing an IntelCore I7-4770 processor with a 3.40 GHz clock and the main memory of 4 GB. The average execution time of the essential security functions is listed in Table 4.

### 7.1. Analysis of Computation Overhead

Instead of communicating with the network server every session to derive a new session key, as mentioned in the basic LoRa protocol, our protocol allows every end device to store n session keys to reduce the communication overhead and save the network bandwidth. Each end device requires initially joining the LoRa network and sharing one session key and one application key. Then it uses these keys to securely share a standard master secret key and a one-way hash function. The end device uses the shared security parameters to generate n hashed keys, n salt values for authentication, and n salt values for encryption. The computation cost for each end device is calculated only one time at the initialization phase or in the key-update process.

- The hash chain generation:
  We assume that hash chain generation can be offline at the initialization phase of joining a new end device to the LoRaWAN network. However, during the runtime, the hash chain generation computation time can be calculated as follows:
  One hash generation using the Sha-256 hash function requires 0.006 ms. According to the LoRaWAN specification [33], in a 24 h interval, a node transmits one packet every 14.4 min. In conclusion, a node sends approximately 11 packets per day. Therefore, the total number of session keys required to secure every transmitted message for 1 year is 3960 keys only. As well, the computation time of the hash chain of a length 5000 key element is 30 ms. Any end device can generate the required keystream during the runtime using the one-way hash function h(.) and the master secret key Sk_n_.
- The salt random number generation:
  Salts are in place to prevent someone from cracking original keys and can be stored in cleartext in the database. We recommend an offline salt generation for each original key to reduce the network overhead. In some cases, such as key updating due to normal situations or under attacks, each end device must generate the salt numbers during runtime. The calculation time of salt numbers includes generating random numbers and XORing of the generated salts and the original keys. End devices must store only the salts numbers, the key indexes, and the salted keys in the hash chain table. Generating different salts results in different keys for both authentication and encryption. For the star topology, which is the dominant topology for LoRaWAN, each end device shares a different master secret key to generate a different hash chain that allows the devices to communicate with the network server and application server securely. In contrast, the end devices in the mesh topology share a common hash chain to communicate with each other and with the network servers. Sharing a single common hash chain can expose the system for sniffing attacks and key compromising attacks. However, the proposed key management scheme allows end devices to locally generate different salt values to hide the original keys, resulting in different salted keys at each device. This salting process makes the breaking of original keys very complex and hides the original keys in a different version of keys, preventing any attacks that target the sniffing of the original keys or the initial master seed key. The total time required for generating n random salts of 8 bytes size required approximately (0.001 n) ms, where a single random number consumes 0.001 ms. For n = 5000, the total computation time is 0.001 × 5000 = 5 ms. The computation time of the XOR operation of the random salt number and the original hashed keys are neglected since their computation time is negligibly short.

Key generation time:

Case 1: for n = 5000 (1 year)

The total computation time of the proposed key generation method at each end device using the crypto functions execution time in Table 3 for n = 5000 keys for 1 year battery lifetime is:

$$\begin{array}{ll} \mathrm{Hash}\;\mathrm{chain}\;\mathrm{time} & \mathrm{generation}+\;\mathrm{salt}\;\mathrm{random}\;\mathrm{generation}\;\mathrm{time}\;\mathrm{for}\;\mathrm{the}\;\mathrm{authentication}\;\mathrm{keys}\\ & +\;\mathrm{salt}\;\mathrm{random}\;\mathrm{generation}\;\mathrm{time}\;\mathrm{for}\;\mathrm{the}\;\mathrm{encryption}\;\mathrm{keys}\;\\ & =N*\;Th+N\;*\;Tg+N\;*\;Tg=5000\left(0.006+0.001+0.001\right)\\ & =40\;\mathrm{ms}. \end{array}$$

Case 2: for n = 417 (1 month)

When the hash chain generation is updated every month, the required number of keys is 417 to allow the end devices to communicate for 1 month. The total computation time for 1 month is:

$$\begin{array}{ll} \mathrm{Hash}\;\mathrm{chain}\;\mathrm{time} & \mathrm{generation}+\;\mathrm{salt}\;\mathrm{random}\;\mathrm{generation}\;\mathrm{time}\;\mathrm{for}\;\mathrm{the}\;\mathrm{authentication}\;\mathrm{keys}\\ & +\;\mathrm{salt}\;\mathrm{random}\;\mathrm{generation}\;\mathrm{time}\;\mathrm{for}\;\mathrm{the}\;\mathrm{encryption}\;\mathrm{keys}\;\\ & =N*\;Th+N\;*\;Tg+N\;*\;Tg=417\left(0.006+0.001+0.001\right)\\ & =3.336\;\mathrm{ms}.\; \end{array}$$

Authentication time:

The computation time required to sign one message is the consumed time to calculate a Message Integrity Code $MIC_{Salted\;k_{Ai}}$ over the message m using a salted authenticated key $Salted\;k_{Ai}.$ One MIC operation using the AES128-CMAC algorithm according to Table 4 is T_MIC_ = 0.0167. The verification time at the receiver (network server) requires one search operation for the key using the attached key index as a pointer and one XORing operation to generate the authenticated salted key, then using the key to generate $MIC_{Salted\;k_{Ai}}^{*}$ value over the same message and comparing the result with the received $MIC_{Salted\;k_{Ai}}$ to accept the message or reject it. Therefore, the total authentication time per message at the end device side is T_MIC_ = 0.0167 ms.

Encryption time:

The computation time required to encrypt one message using AES-128 and a salted encryption key $Salted\;k_{Ei}$ requires T_enc_ = 4.0274ms. The decryption process at the application server side requires one search operation to find the key and XORing the key with the received encrypted salt value to generate the encrypted salt key $Salted\;k_{Ei}$ that was used to decrypt the received message. We neglect the search operation and XORing operation due to their short computation time. Therefore, the total encryption time per message at the end device is T_dec_ = 4.1524. We summarize the security computation overhead of the proposed protocol in Table 5.

### 7.2. Analysis of Communication Overhead

**Figure 14:**
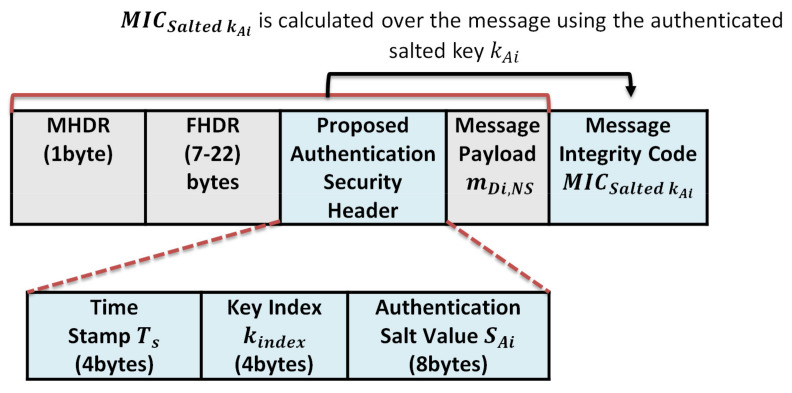
The proposed protocol authenicated message format.

**Figure 15:**
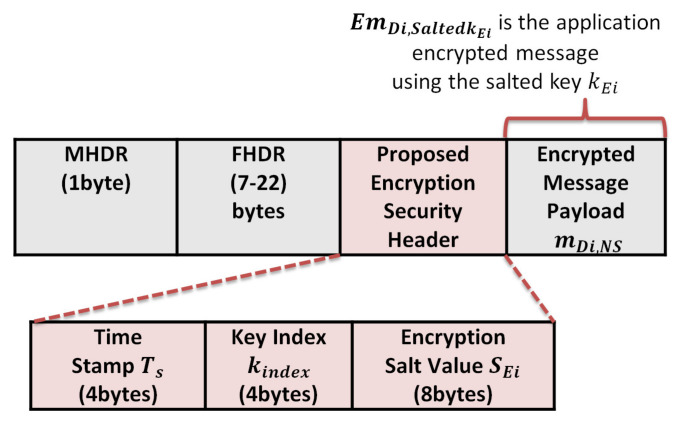
The proposed protocol encrypted message format.

This section analyzes the communication cost in terms of message size for the authenticated messages between the end devices and the network server, the encrypted messages between the end devices and application server, and the required storage at each end device for storing the keystream of length n. The message format for the authenticated messages and encrypted messages are shown in Figure 14 and Figure 15, respectively.

Case 1: High-security level for low power consumption

First: The initial storage overhead at the end devices is calculated as follows:

- For hash chain length n = 5000, each hashed element represents an original key with size 32 bytes, so the total hash chain storage is 5000 × 32 = 160,000 bytes. However, the key size is 32 bytes; we use only 16 bytes key size to support AES-128 authentication and encryption;
- For n salt values used for generating the authentication keys, each salt size is 8 bytes, and the total size is 5000 × 8 = 40,000 bytes;
- For n salt values used for encryption, each salt size is 8 bytes; the total size is 5000 × 8 = 40,000 bytes;
- For n key indices that are used as a pointer for each key in the hash table, each key index is 4 bytes, so the total key indices size is 5000 × 4 = 20,000 bytes.

After generating the salted authentication keys and salted encryption keys, each end device only stores the salted keys, salt values, and key indices. To avoid the physical attacks and prevent any exposure to the original hashed keys, the end device removes all original hashed keys and only keeps the salted authentication and encryption keys.

The total final storage is calculated for n = 5000 as follows:

$$\begin{array}{ll} n\;\mathrm{key}\;\mathrm{index}\;+\;n & \mathrm{salt}\;\mathrm{value}\;\mathrm{for}\;\mathrm{authentication}\;+\;n\;\mathrm{salt}\;\mathrm{value}\;\mathrm{for}\;\mathrm{encryption}\;\\ & +\;n\;\mathrm{salted}\;\mathrm{authentication}\;\mathrm{keys}\;+\;n\;\mathrm{salted}\;\mathrm{encryption}\;\mathrm{keys}\;\\ & =n*4+n*8+n*8+n*16+n*16\\ & =5000\left(4+8+8+16+16\right)=260,000\;\mathrm{bytes} \end{array}$$

Second: the security overhead of the authenticated message and encrypted message:

- The security overhead in the case of authentication is calculated as follows:
  The structure of the authentication message is shown in Figure 9 and consists of the following elements $\left(MHDR\left|\left|FHDR\right|\right|T_{s}\left|\left|k_{index}\right|\left|S_{Ai}\right||m_{Di,NS}\right||MIC_{Salted\;k_{Ai}}\right)$. We exclude the MAC header (MHDR), Frame header (FHDR), and the message payload $m_{Di,NS}$ during calculation. So the total communication overhead due to security header and signature per message is 4 + 4 + 8 + 4 = 20 bytes;
- The security overhead in the case of the encrypted message is calculated as follows:
  The message structure of the encrypted message is shown in Figure 10 and consists of the following elements: $\left(MHDR\left|\left|FHDR\right|\right|T_{s}||k_{index}||S_{Ei}||Em_{Di,Salted\;k_{Ei}}\right).$ We exclude the MAC header (MHDR), Frame header (FHDR), and the encrypted message payload $Em_{Di,Salted\;k_{Ei}}$ during calculation, so the total communication overhead due to security per message is 4 + 4 + 8 = 16 bytes.

Case 2: Medium security-level for extremely low power consumption

- By allowing the end devices to update the stored keys every month, once the required keys per year for standard LoRaWAN networks are 5000 keys, each end device needs to store only 417 keys for 1 month of communication.
- Due to the IoT devices’ memory and power limitation, the security overhead can be decreased using salt values of size 2 bytes and the key index of size 2 bytes, so the required storage is $\left\{n\;\mathrm{key}\;\mathrm{index}\;+\;n\;\mathrm{salt}\;\mathrm{value}\;\mathrm{for}\;\mathrm{authentication}\;+\;\;n\;\mathrm{salt}\;\mathrm{value}\;\mathrm{for}\;\mathrm{encryption}\;+\;n\;\mathrm{salted}\;\mathrm{authentication}\;\mathrm{key}\;+\;\;n\;\mathrm{salted}\;\mathrm{encryption}\;\mathrm{key}\right\}=n*2+n*2+n*2+n*16+n*16=417\left(38\right)=\;15,846\;\mathrm{bytes}$.
- Using a timestamp of 2 bytes, the security overhead in the case of message authentication is 2 + 2 + 2 + 4 = 10 bytes. In the case of an encrypted message, the overhead is 2 + 2 + 2 = 6 bytes.

We summarize the security overhead of the proposed protocol in Table 6.

### 7.3. Power Consumption Analysis

In this section, we analyze the required power consumption of the end devices due to the security overhead. We used the LoRa energy calculator [34] to calculate the end device’s battery lifetime under defined assumptions and different packet sizes using real-world measurements. The inputs to the LoRa energy calculator are packet size, transmission period, and battery type. The measured outputs are the time on the air, the number of transmitted packets, and the Time To Live (TTL) of the end device battery. To demonstrate the differences between the proposed protocol and other related LoRaWAN protocols, we choose also the enhanced LoRaWAN protocol proposed by You et al. [19]. In [19], the authors proposed an enhanced protocol of LoRaWAN using Elliptic Curve Diffie Hellman (ECDH) key generation, which is authenticated by Elliptic Curve Digital Signatures. Their protocol supports end-to-end security and suggests the generation of DevNonce to avoid replay attacks. In [19], the authors proposed two different cases for their method, the first one is the Default Option (DO) and the second one is the Security-Enhanced Option (SEO). Both options are to prevent the network server from generating any vulnerabilities and breaking the security between the end node device and the application server. The first case DO targets to defend against network server eavesdropping attacks that attempt to break the communication security between the end device and its related application server. The second SEO case prevents the data manipulating between the end device and application server and also prevents the impersonation of both entities. The malicious network server is blocked from manipulating packets between a device and its application server, as well as impersonating these two parties. However, although this protocol supports the LoRaWAN end-to-end security, it suffers from high computation cost and high communication cost due to using expensive elliptic curve operations. In this section, we analyze the original LoRaWAN protocol, the proposed protocol, and both cases of enhanced LoRaWAN mentioned in [19]. During the performance evaluation process, we used a Li-ion(1000 mAh, 3.3 Volt) battery that consumes a processing power of 15 mW for reading a sensor value within a 5 ms period, and the sleep consumption is 10uW. The packet transmission is periodic every 14.4 min according to the LoRaWAN standard. LoRaWAN communication supports multiple spread factors (SF) (between 6 and 12) to compromise the data transfer rate and the communication range. Each node transmits every 14.4 ms, approximately 11 packets are transmitted every day. We analyze the protocol of [19] using the LoRa energy calculator under defined assumptions and different packet sizes using real-world measurements. Table 1 shows the results for time on the air, the number of transmitted packets, and the battery life during the communication. The original LoRaWAN MAC payload size ranges between 51 and 222 bytes. We added the security overhead in terms of bytes to the original payload size to study the security impact on the battery lifetime. The total communication cost and computation cost for the Do case are 94 bytes and 11 ms, respectively. 

**Figure 16:**
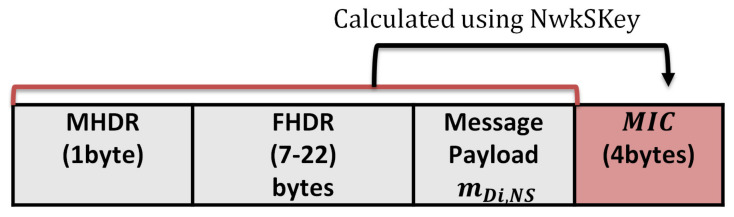
The original message format of LoRaWAN.

Table 7 lists the main parameters of the LoRa communication protocol, such as the spreading factor, channel bandwidth, and the transmitted power. The SF is the modulation technique that represents the number of chips per symbol. It is an integer value between 6 and 12: the greater the SF value, the more capable the receiver is to move away from the signal noise. Therefore, the greater the SF value, the more time is required to transmit a packet. The channel bandwidth represents the range of transmission band frequencies [35]. The channel BW can be 125 kHz, 250 kHz, or 500 kHz. For a fast transmission, a 500 kHz BW is recommended. We used a 125 kHz channel BW to support a long-wide communication. We studied the power consumption using two cases (worst case and best case). The worst case supports SF12, the channel BW 125 kHz, and the transmission power is 14 dBm. The best case supports SF7, a channel BW of 125 kHz, and a transmission power of 2 dBm.The original message format of the LoRaWAN for an authenticated message is as follows: The LoRaWAN message format that consists of the MAC header (MHDR), Frame header (FHDR), message payload from end device to the server (m), and the Message Integrity Code (MIC), as shown in Figure 16. The power analysis for the original LoRaWAN is calculated for message payload sizes $m_{Di,Ns}$ 51,136, and 222 bytes using the LoRaWAN parameters in Table 7 and excluding the message headers. To study the impact of security on power consumption, a 4 bytes MIC is included for authentication for each transmitted packet. From Table 8, the measured parameters for the original LoRaWAN are time on the air, the number of packets, and Time To Live (TTL). Time on the air represents the total transmission time for this end device during the battery life.

In LoRaWAN, for a Spreading Factor (SF) of 12 (worst case) and payload size of 51 bytes, the time on the air is 2465 ms, while for payload size, 222 is 8036 ms. The time on the air for a maximum LoRaWAN payload size of 222 bytes is increased by 3 times more than the minimum LoRaWAN payload size of 51. In contrast, the total transmitted packets for an SF of 12 is 32,339 packets with a payload size of 51 bytes, while for a payload size of 222, the number of transmitted packets is 10847. The total transmitted packets are decreased by 3 times for the maximum payload size of 222 bytes. The Time To Live for an SF 12 for the original LoRaWAN with a payload size of 51 bytes is 0.8 years, while for a payload size of 222 bytes, the battery’s lifetime is dropped dramatically for 0.2 years. We conclude that the worst-case scenario for the LoRaWAN with SF (12), the greater packet size, and the shorter battery lifetime.

The results for the LoRaWAN in the best-case scenario with an SF of 7 for a payload size 51, 136, and 222 bytes are also shown in Table 7. For a payload size of 51 bytes, the time on the air is 102 ms, the transmitted packets are 267664, and the battery life is 7.3 years. For a payload size of 222 bytes, the time on the air is 353 ms, the transmitted packets are 182,980 packets, and the battery lifetime is 4.9 years. We conclude the results of LoRaWAN for the best case that the battery lifetime is dropped by 3 years for the maximum payload size of 222.

The battery life analysis of the proposed protocol low power case and extremely low power case are shown in Table 9 and Table 10, respectively. From Table 9, the proposed protocol low power authentication is analyzed with a security overhead of 20 bytes to each transmitted payload. For an SF of 12 (worst case) and payload size of 51 bytes, the time on the air is 2957 ms, the transmitted packets are 27,527, and the TTL is 0.7 years. In contrast to the payload size of 222 bytes, the time on the air is 8527 ms, the transmitted packets are 10,246, and the TTL is 0.26 years. For the SF of 12, when the packet size increased by more than 3 times, the battery life dropped by 3 times.

For an SF of 7 (best case) and a payload size of 51 bytes, the time on the air is 128 ms, the transmitted packets are 255,593, and the TTL is 6.9 years. For a maximum packet size of 222 bytes, the proposed protocol can support a time on the air of 374 ms, the total transmitted packets are 178,373, and the TTL is 4.8 years. For the SF of 7, when the packet size increased by 3 times, the battery life dropped by one time.

In the same way, the proposed protocol for extremely low power authentication with a security overhead of 10 bytes is illustrated in Table 10. From Table 10, we prove that decreasing the security overhead from 20 bytes to 10 bytes enhances the overall performance and extends the battery life.

Table 11 and Table 12 show the battery analysis for [19] options (DO, SEO). In the same way, we analyze the You et al. protocol cases in terms of Time on the air, Number of packets, and the Time to Live in years. Table 11 shows the results for the DO option for security overhead 94 bytes using the elliptic Curve Diffie Hellman (ECDH) key exchange that introduces very high computation cost and communication cost as explained previously. Under the same environment and assumptions, we measured the options of [19] for different packet sizes (51,136 and 222) bytes. For the SEO option when the security is enhanced with 126 bytes overhead, the battery life is decreased, and time on the air is increased compared with the DO option, in which the security overhead is only 94 bytes.

**Figure 17:**
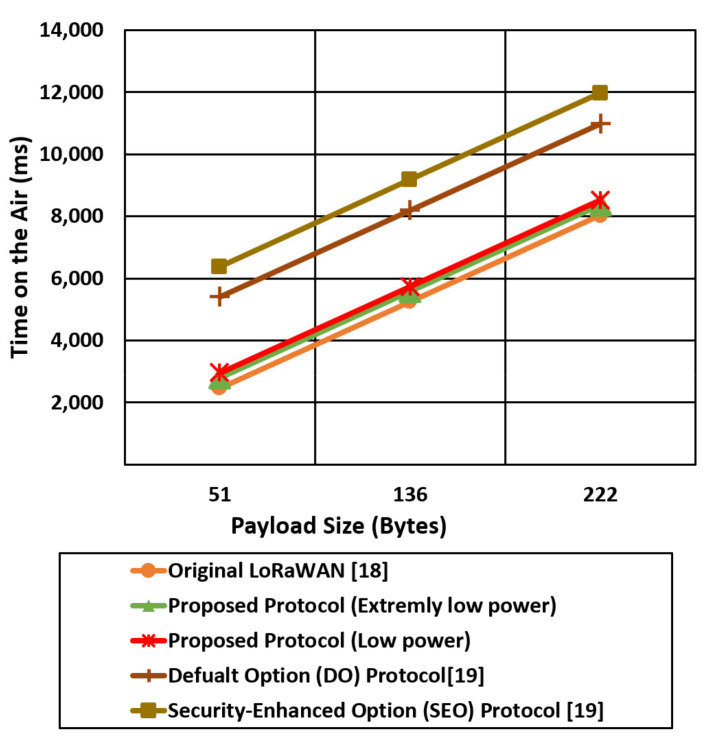
The time on the air comparison for the proposed protocol cases and the other mentioned protocols using the worst-case environment parameters (SF 12).

**Figure 18:**
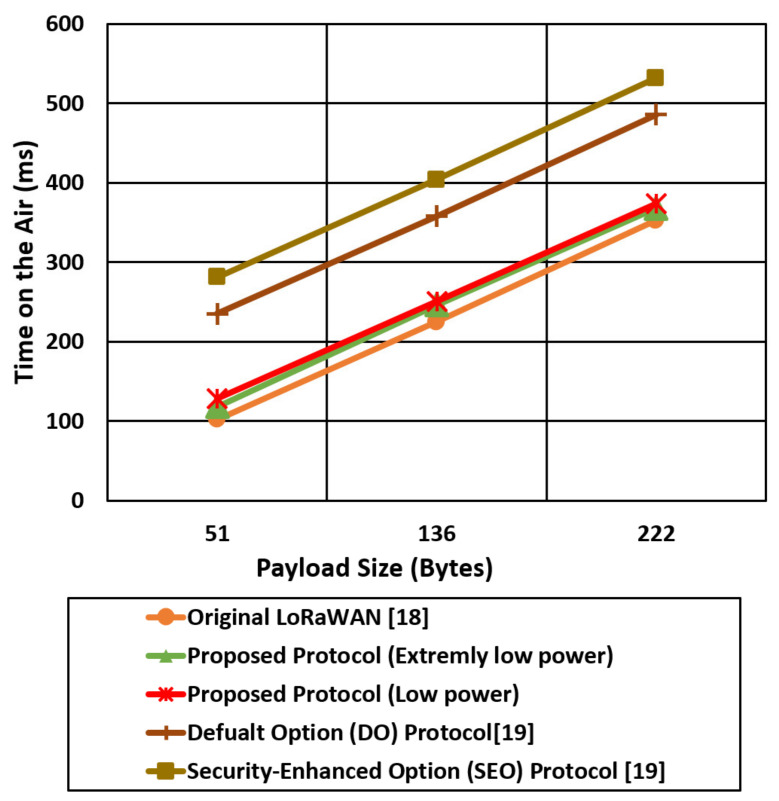
The time on the air comparison for the proposed protocol cases and the other mentioned LoRaWAN protocols using the best-case environment parameters (SF 7).

**Figure 19:**
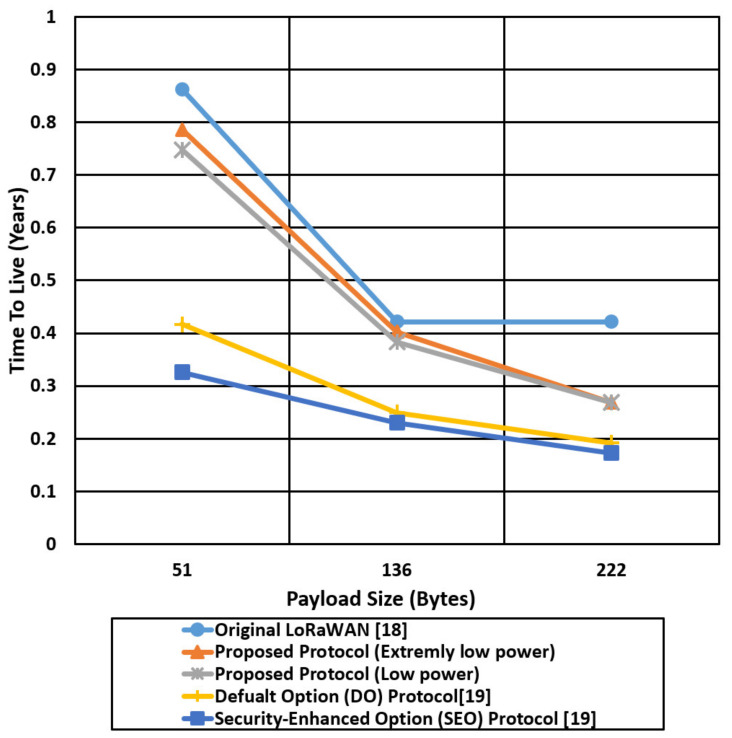
Comparison of the battery lifetime in terms of years for the proposed protocol cases and the other mentioned protocols using the worst-case environment parameters (SF 12).

**Figure 20:**
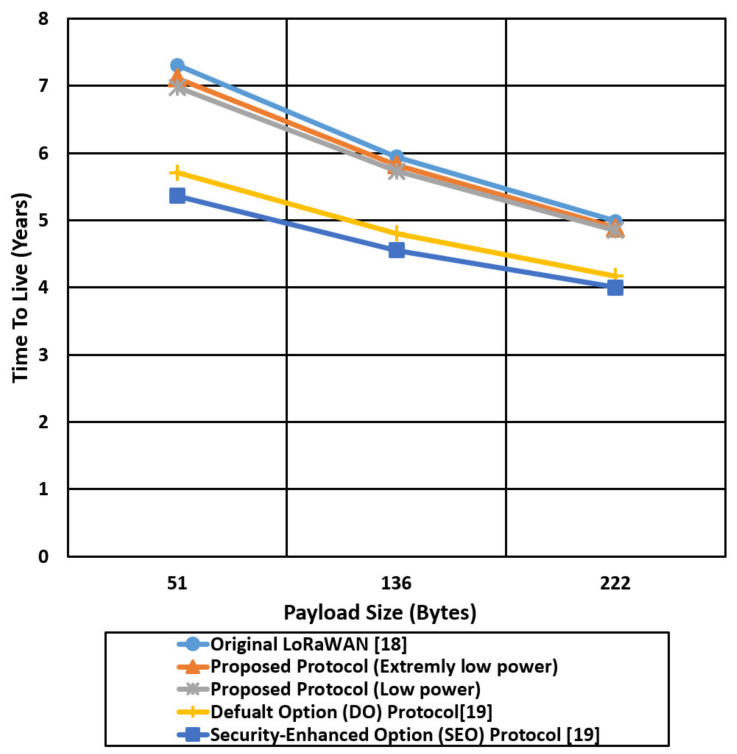
Comparison of the battery lifetime in terms of years for the proposed protocol cases and the other mentioned protocols using the best-case environment parameters (SF 7).

We compare the proposed protocol (low power authentication and extremely low power authentication) with the other related LoRaWAN mentioned protocols in terms of time on the air and Time To Live (TTL) for different packet sizes (51,136,222) bytes, as shown in Figure 17, Figure 18, Figure 19 and Figure 20.

Figure 17 shows the time on the air comparison for the proposed two cases against the original LoRaWAN and the two DO and SEO cases of [19] using an SF of 12. The time on the air for the mentioned protocols is increased linearly with the payload size, while the LoRaWAN experiences the lowest time on the air compared with the proposed protocol cases (low power, extremely low power) and [19] cases. Figure 17 also shows that Do and SEO protocols introduce very high transmission time (time on the air) compared with the proposed protocol and standard LoRaWAN. SEO introduces very high time on the air due to high-security overhead that reaches approximately 126 bytes. The proposed protocol extremely low power case reducing the time on the air by 36%, 26% compared with SEO and DO, respectively, for a packet size of 222 bytes using an SF of 12.

However, reducing the security overhead in extremely low power cases reduces the time on the air compared with the low power cases that extend the battery lifetime.

Figure 18 shows the time on the air comparison for the proposed security cases (low power, extremely low power) and the other LoRaWAN protocols (original, Do, SEO) using an SF of 7. The spread factor value of 7 allows the end devices to reduce the time on the air and save the power consumption compared with spread factor 12. The LoRaWAN original protocol experiences the lowest time on the air compared with the other protocols since the total security overhead for the LoRaWAN is 4 bytes. The proposed extremely low power case reduced the time on the air by two times compared with the SEO case. The end devices in SEO use a complex and expensive elliptic curve to provide authentication that introduces high power consumption and high time on the air.

Figure 19 compares the battery life in years of the proposed protocol in two cases and the other mentioned protocols for different payload sizes (51,136,222) bytes using the worst-case environment parameters (SF of 12). The battery life (Time To Live) is decreased dramatically with the increase of the payload size; for the LoRaWAN protocol, the TTL of an end device is 0.88 years for a payload size of 51 bytes, compared with 0.72 years and 0.78 years for the proposed protocol cases (low power, extremely low power), respectively. In contrast, for a payload size of 222 bytes, the TTL for LoRaWAN is decreased from 0.88 to 0.29 years, and for the proposed protocol is decreased from 0.72, 0.78 to 0.27, 0.28 for the low power case and extremely low power case, respectively. The proposed protocol for the extremely low power case enhances the battery life of the end devices by two times over the SEO protocol for a payload size of 222 bytes due to using a negligible hash chain key generation compared with the digital certificate authentication that is used in the SEO protocol.

Figure 20 shows the TTL comparison of the proposed protocol cases and other compared protocols using a Spread Factor of 7 that extends the battery life to a couple of years, compared with the worst-case scenario with SF 12. For the best-case scenario of SF 7, the battery life is extended to 7 years in LoRaWAN for a payload size of 51 and decreased to 5 years for a payload size of 222 bytes. The extremely low power protocol case extends the battery life by two times compared with the SEO protocol for a payload size of 222 bytes. SEO introduces 126 bytes security overhead to establish end-to-end security between the end device and network server that extends the time on the air and consumes the battery power very quickly.

The advantages of the proposed protocol are summarized as follows:

- The proposed key management protocol introduces a little computation overhead compared with the original LoRaWAN to enhance the security by generating salts random values and n authentication keys;
- The two proposed cases outperform the SEO and DO protocols in terms of time on the air and battery life;
- The proposed key management protocol uses a negligible hash chain generation that supports security with low power consumption;
- The proposed protocol uses salt encryption that hides the originally generated keys in different forms and prevents physical attacks;
- The proposed protocol protects the network from replay attacks by including a fresh timestamp per each transmitted message;
- For various IoT applications, where high-security levels and battery lifetime are traded off, our protocol can be configured as demanded. For example, test results for two cases (low power, extremely low power) are presented;
- The proposed protocol can be implemented in extremely low power mode. For example, case 2 can further reduce the power consumption over case 1 by reducing the security overhead from 20 bytes to 10 bytes;
- The proposed protocol enhances the security level of the IoT networks at a little sacrifice in power consumption.

## 8. Conclusions and Future Work

This paper proposed a key management protocol for LoRaWAN networks that can support session key updates and defense against key attacks. One master secret key is used to generate n secret keys using a one-way hash function h(.). Salt encryption is supported for the generated hashed keys to protect them against the physical attacks in contrast to the LoRaWAN vulnerable to key compromising attacks. This paper presented two case studies for the key management protocol: one case supporting higher security with low power and the other case supporting extremely low power. We compared the two cases against the original LoRaWAN and other related protocols regarding power consumption and time on the air. The proposed cases demonstrated significant security enhancement at the cost of negligible overhead in the power consumption compared with the original LoRaWAN. In addition, the proposed extremely low power case reduces the power consumption by two times compared with SEO protocol [19] for a packet size of 222 bytes. The proposed protocol extremely low power case reduced the time on the air by 36%, 26% compared with SEO and DO, respectively, for a packet size of 222 bytes using an SF of 12. The proposed protocol, therefore, is well suited to low power applications that require a higher security level with reliable periodic key updates. We continue this work by defining new types of attacks that target the LoRaWAN networks. We intend to verify the security level using automatic verification analysis. We also extend the simulation environment to a real testbed using real LoRaWAN devices to have accurate results concerning the power consumption and transmission time. In addition, we plan to use machine learning techniques in training IoT data and classify it against well-known attacks. We plane to propose a security solution for LoRaWAN applications within 5G networks [36,37]. 

## Figures and Tables

**Table 1:** Comparison between the existing LoRaWAN key management protocols and the proposed protocol.

Security Functions	LoRaWAN [18]	You et al. [19]	Butun et al. [20]	Naoui et al. [22]	Girard et al. [23]	The Proposed Protocol
Mutual Authentication	√	√	√	√	√	√
Session Key Updates	×	×	×	×	√	√
Message Integrity	√	√	√	√	√	√
Message Encryption	√	√	√	√	√	√
Perfect Forward Secrecy	×	√	×	√	×	√
Secure Key Exchange	×	×	×	×	×	√
Defense Against Physical Key Attacks	×	×	×	×	×	√
Defense Against Replay Attacks	×	×	×	×	×	√

**Table 2:** The system abbreviations and notations.

Notations	Descriptions
OTAA	Over-The-Air Activation
NwkSKey	Network session key
MIC	Message integrity code
Join-Request	Join-Request to attach the end device to the LoRa network
h(.)	One-way hash function of length n
DevNonce	Nonce value randomly generated by the device
DevEU I	Device identifier
CMAC	Cipher-based message authentication code
AppSKey	Application session key
AppNonce	Nonce value randomly generated by the network server
AppKey	The long-term key shared between a device and a network server
AppEU I	Application identifier
AES	Advances Encryption Standard
ABP	Activation By Personalization
$T_{s}$	The current timestamp to avoid replay attacks
${\mathrm{Sk}}_{n}^{u}$	The updated master secret seed of length n
${\mathrm{Sk}}_{n}$	The master secret key to that iteratively hashed n times to generate a keystream of length n
$S_{\mathrm{Ei}}$	The randomly generated salted value is used to hide the encryption key
${\mathrm{Salted}\;k}_{\mathrm{Ei}}$	An encrypted Salted encryption key ${\mathrm{Salted}\;k}_{\mathrm{Ei}}$ = ($K_{i}$⊕$S_{\mathrm{Ei}}$)
${\mathrm{Salted}\;k}_{\mathrm{Ai}}$	An encrypted Salted authentication key ${\mathrm{Salted}\;k}_{\mathrm{Ai}}$ = ($K_{i}$⊕$S_{\mathrm{Ai}}$)
$S_{\mathrm{Ai}}$	The randomly generated salted value is used to hide the authentication key
${\mathrm{MIC}}_{{\mathrm{Salted}\;k}_{\mathrm{Ai}}}$	The MIC value for message m using a salted authentication key of ${\mathrm{Salted}\;k}_{\mathrm{Ai}}$
$m_{\mathrm{Di},\mathrm{NS}}$	The transmitted message from the end device Di to the network server Ns
$k_{i}$	One of the generated hashed keys that generated by iteratively hashing the master secret seed ${\mathrm{Sk}}_{n}$
$k_{\mathrm{index}}$	The pointer of the used key in authentication in the pre-shared common hash table

**Table 3:** The security comparison of the proposed key management protocol with the original LoRaWAN.

Security Functions and Defense against Attacks	The Original LoRaWAN Protocol [17]	The Proposed Protocol
Session Key Distribution	The end device shares two-lifetime session keys with the network server; Network server suffers from high frequent communication with the end devices for deriving new session keys; The end devices suffer from high power consumption and key compromising attacks due to using two long-life keys.	Network server considered as trusted third-party authority; A network server, application server, and end device share a common hash chain of n keys; No frequent communication with the network server for key establishment; MAC layer messages and application layer messages are protected with different hashed keys; Enhances the security level and prevents the use of two long-life session keys.
Mutual Authentication	Only the initial join procedure authentication to generate two session keys NwkSKey and AppSKey.	Using the initial join procedure to generate NwkSKey and AppSKey; End devices securely share an encrypted master secret key (Skn) with the network server to generate a hash chain for future session communications.
Session Key Updates	The key update is not supported; Only two longlife keys are used for different sessions.	Support key update; Using different session keys with each message.
Message Integrity	Supported: Using AES128-CMAC	Supported: Using AES128-CMAC
Message Encryption	Supported: Using AES128-CTR	Supported: Using AES128-CTR
Perfect Forward Secrecy	Not supported: Compromising the root keys can compromise the session keys.	Supported: Compromising the root keys cannot compromise the encrypted session keys. Salted keys are stored at the end device side.
Secure Key Exchange	Not supported: An attacker can sniff the transferred parameters in the initial join procedure and derive the session keys.	Supported: The master secret key that is used to generate the hash chain elements is shared in an encrypted way.
Defense against key-compromising Attacks	Not supported: No defense against the key attacks. As the root keys and session keys can be sniffed and derived.	Supported: Can defend against the key attacks using a salted key table that hides the original keys in a different encrypted form. Hiding keys prevent attackers from sniffing it or even compromise.
Defense against replay attacks	Not supported: No defense against the replay attacks as the end device does not register the received AppNonce in the Join-Accept message.	Supported: Can defend against the replay attacks by attaching a fresh timestamp with each message.

**Table 4:** The definition and processing time of the primary cryptographic operations of NS3 Simulator for the proposed method.

Cryptographic Operation	Definition and Abbreviation	Average Execution Time (ms)
Message Hashing	T_h_: the time defined for one hash function operation using SHA-256 algorithm	0.006
Message Encryption	T_enc_: the time to perform one encryption operation using AES-128	4.0274
Message Decryption	T_dec_: the time to perform one decryption operation using AES-128	4.1524
Random number generation	T_g_: the time required to generate one random number	0.001
Message integrity coding	T_MIC_: the time defined for one MIC operation using AES128-CMAC algorithm	0.0167

**Table 5:** The compution time calculation of the proposed key mangment protocol at the end device.

Compution Time (ms)		
Required Key generation time	Case 1 (n = 5000 key)	Case 2 (n = 417 key)
	40	3.336
Required Authentication time per message	0.0167	
Required Encryption time per message	4.0274	

**Table 6:** The Security overhead of the proposed key mangment protocol at the end device.

Security Overhead (Bytes)	Case 1 (n = 5000 Key)	Case 2 (n = 417 Key)
Storage size	260,000	15,846
Authentication overhead size	20	10
Encryption overhead size	16	6

**Table 7:** LoRaWAN paramaters.

Parameters Assumptions	Worst Case	Best Case
Spreading Factor (SF)	12	7
Channel Bandwidth (BW)	125 kHz	125 kHz
Transmission Power (dBm)	14	2

**Table 8:** Battery lifetime in the case of the original LoRaWAN with authentication for different message sizes.

Parameters	Payload Size = 51 Bytes		Payload Size = 136 Bytes		Payload Size = 222 Bytes	
	Worst Case	Best Case	Worst Case	Best Case	Worst Case	Best Case
Time on the air (ms)	2465	102	5251	225	8036	353
Number of packets	32,339	267,664	16,245	218,202	10,847	182,980
TTL (Time To Live) (Years)	0.863014	7.30685	0.421918	5.94521	0.287671	4.9863

**Table 9:** Battery lifetime in the proposed protocol for low power authentication case 1 (16 bytes overhead due to secuirty).

Parameters	Payload Size = 51 Bytes		Payload Size = 136 Bytes		Payload Size = 222 Bytes	
	Worst Case	Best Case	Worst Case	Best Case	Worst Case	Best Case
Time on the air (ms)	2957	128	5742	251	8527	374
Number of packets	27,527	255,593	14,933	210,113	10,246	178,373
TTL (Time To Live) (Years)	0.747945	6.98082	0.383562	5.73425	0.268493	4.85205

**Table 10:** Battery lifetime in the case of the proposed protocol extremly low power authentication case 2 for different message sizes (10 bytes overhead due to secuirty).

Parameters	Payload Size = 51 Bytes		Payload Size = 136 Bytes		Payload Size = 222 Bytes	
	Worst Case	Best Case	Worst Case	Best Case	Worst Case	Best Case
Time on the air (ms)	2793	118	5578	246	8364	368
Number of packets	28,963	260,289	15,346	211,683	10,439	179,503
TTL (Time To Live) (Years)	0.786301	7.11791	0.40274	5.82611	0.268493	4.89041

**Table 11:** Battery lifetime in the case of [19] Defualt Option (DO) case for different message sizes (94 bytes overhead due to security).

Parameters	Payload Size = 51 Bytes		Payload Size = 136 Bytes		Payload Size = 222 Bytes	
	Worst Case	Best Case	Worst Case	Best Case	Worst Case	Best Case
Time on the air (ms)	5414	235	8200	358	10,985	486
Number of packets	15,787	215,588	10,640	182,304	8025	157,047
TTL (Time To Live) (Years)	0.416667	5.709589	0.249315	4.805479	0.191781	4.172603

**Table 12:** Battery lifetime in the case of [19] Security-Enhanced Option (SEO) for different message sizes (126 bytes overhead due to secuirty).

Parameters	Payload Size = 51 Bytes		Payload Size = 136 Bytes		Payload Size = 222 Bytes	
	Worst Case	Best Case	Worst Case	Best Case	Worst Case	Best Case
Time on the air (ms)	6397	281	9183		11,968	532
Number of packets	13,485	201,774	9543	172,327	7384	149,587
TTL (Time To Live) (Years)	0.326027	5.364384	0.230137	4.556164	0.172603	4
